# YAK577 Attenuates Vascular Calcification by Targeting an MMP14–NOX2/ROS Axis in VSMCs and a Vitamin D_3_-Induced Mouse Model

**DOI:** 10.3390/antiox15050605

**Published:** 2026-05-10

**Authors:** Hongyan Zhou, Hae Jin Kee, Seong Min Jeong, Liyan Bai, Le Wan, Seong Hoon Kim, Seung Hun Lee, Thomas Kurz, Doo Sun Sim, Myung Ho Jeong, Young Joon Hong

**Affiliations:** 1 Heart Research Center, Chonnam National University Hospital, Gwangju 61469, Republic of Korea; zhouhy202111@gmail.com (H.Z.); wakeupmr@nate.com (S.M.J.); 2 Hypertension Heart Failure Research Center, Chonnam National University Hospital, Gwangju 61469, Republic of Korea; 3 Biomedical Research Institute, Chonnam National University Hospital, Gwangju 61469, Republic of Korea; 4 Emergency Critical Center, Beijing Anzhen Hospital, Capital Medical University, Beijing 100029, China; bailiyan@naver.com; 5 Department of Orthopedics, Chonnam National University Hospital, Gwangju 61469, Republic of Korea; wle202302@gmail.com; 6 Department of Cardiology, Chonnam National University Medical School, Gwangju 61469, Republic of Korea; kimtae1998@naver.com (S.H.K.); ish8602@naver.com (S.H.L.); true1021@naver.com (D.S.S.); 7 Department of Biomedical Sciences, Graduate School of Chonnam National University, Hwasun 58128, Republic of Korea; 8 Institute of Pharmaceutical and Medicinal Chemistry, Heinrich Heine University Düsseldorf, Universitätsstrβe 1, 40225 Düsseldorf, Germany; thomas.kurz@hhu.de; 9 Gwangju Veterans Hospital, Gwangju 62284, Republic of Korea; myungho6243@naver.com

**Keywords:** YAK577, vascular calcification, histone deacetylase inhibitor, MMP14, NOX2, NOX2-derived ROS, osteogenic transdifferentiation, vascular smooth muscle cells, vitamin D_3_-induced vascular calcification

## Abstract

Vascular calcification is an actively regulated process driven by vascular smooth muscle cell (VSMC) osteogenic reprogramming and promoted by oxidative stress and extracellular matrix remodeling. We investigated whether the novel histone deacetylase inhibitor YAK577 mitigates calcification by modulating an MMP14–NOX2/ROS-associated pathway in calcification medium (CM)-treated VSMCs and a vitamin D_3_-induced arterial calcification model in 8-week-old male C57BL/6N mice. Calcification was assessed by Alizarin Red S/von Kossa staining and calcium quantification; osteogenic markers (BMP2, RUNX2, MSX2) and MMPs were examined by qRT-PCR and immunoblotting; intracellular ROS was measured by DHE staining with N-acetylcysteine as an antioxidant control; and MMP14 was manipulated by siRNA knockdown or plasmid overexpression. YAK577 was non-cytotoxic at effective concentrations and reduced CM-induced calcium deposition and osteogenic marker expression. YAK577 reduced MMP14 expression and suppressed CM-induced NOX2/p47phox activation and ROS accumulation, while GSK2795039 attenuated CM-induced DHE fluorescence. MMP14 silencing attenuated, whereas MMP14 overexpression enhanced, osteogenic signaling and increased NOX2. In vivo, YAK577 reduced vitamin D_3_-induced aortic calcium burden, histological calcification, and the expression of MMP14, NOX2, and osteogenic markers. These data support a working model in which YAK577 alleviates vascular calcification, at least in part, by suppressing an MMP14-associated NOX2/p47phox–ROS axis.

## 1. Introduction

Vascular calcification is a frequent and clinically important complication of cardiovascular and metabolic diseases, including atherosclerosis, diabetes, and chronic kidney disease [1,2,3]. Once regarded as a passive precipitation of minerals, it is now understood as an actively regulated process in which calcium–phosphate deposits accumulate within the vessel wall and are accompanied by cellular and molecular changes reminiscent of bone formation [4,5,6]. In particular, vascular smooth muscle cells (VSMCs) can undergo osteogenic transdifferentiation, characterized by induction of osteogenic regulators such as bone morphogenetic protein-2 (BMP2), runt-related transcription factor-2 (RUNX2), and msh homeobox-2 (MSX2) [7,8,9,10]. This phenotypic shift is often accompanied by impaired anti-calcific defenses, including altered activity of endogenous inhibitors such as matrix Gla protein and fetuin-A [5,6]. The osteogenic conversion of VSMCs contributes to arterial stiffening and is associated with increased cardiovascular morbidity and mortality; however, effective pharmacological strategies that directly target vascular calcification remain limited, underscoring the need to define actionable mechanisms and identify druggable nodes.

A major theme in the field is that vascular calcification is driven by a convergence of extracellular matrix (ECM) remodeling, inflammatory signaling, and oxidative stress. Reactive oxygen species (ROS) not only amplify inflammation but also promote osteogenic gene programs and mineral deposition in VSMCs [11]. NADPH oxidases, particularly NOX2, represent key enzymatic sources of vascular ROS [12]. NOX2 activity is regulated not only by NOX2 protein abundance but also by activation-dependent assembly of cytosolic subunits, among which p47phox is a central organizer whose phosphorylation promotes formation of the active oxidase complex [13,14]. Despite broad agreement that oxidative stress accelerates calcification, a key unresolved question is how redox pathways are mechanistically linked to the matrix-remodeling events that characterize calcifying vessels. Addressing this gap is especially relevant to *Antioxidants*, because it may reveal therapeutic opportunities that simultaneously suppress oxidative injury and calcific remodeling.

Matrix metalloproteinases (MMPs) are central mediators of ECM turnover in vascular disease and have increasingly been implicated in calcification [15,16,17]. Several MMPs have been associated with calcific phenotypes: for example, MMP3 has been reported to promote medial calcification through osteogenic conversion of VSMCs [18]. MMP2 and MMP9 are prominent during atherosclerosis development in Ldlr(-/-)Apob(100/100) mice [19], and both enzymes have also been implicated in uremic vascular calcification [16]. MMP10 has been linked to calcification via AKT-related signaling in valve disease [20]. Among membrane-type MMPs, MMP14 (MT1-MMP) is of particular interest because it controls pericellular proteolysis and protease cascades and is widely recognized as an activator of pro-MMP2 [15,21], and has been implicated in VSMC phenotypic modulation through LRP1 processing [22]. Yet, whether MMP14 acts as a mechanistic hub connecting ECM remodeling to oxidative signaling during vascular calcification remains insufficiently defined.

Epigenetic regulation provides another layer of complexity and opportunity. Histone deacetylases (HDACs) modulate transcriptional programs relevant to inflammation, fibrosis, and vascular remodeling by deacetylating histone and non-histone proteins [23,24,25]. HDAC inhibitors have attracted attention as therapeutic agents, but their effects on vascular calcification are controversial and appear to depend on isoform selectivity and experimental context. For example, the pan-HDAC inhibitor trichostatin A has been reported to promote phosphate-induced VSMC calcification [26], and disruption of HDAC1 regulation has been linked to enhanced vascular calcification [27]. In contrast, genetic evidence suggests that HDAC9 contributes to aortic calcification and influences VSMC phenotype in atherosclerosis-relevant settings [28]. This divergence highlights a major need: to identify HDAC-modulated pathways that can be therapeutically leveraged to suppress calcification without triggering pro-calcific side effects.

YAK577 is a novel hydroxamic acid-based HDAC inhibitor that we previously reported to improve cardiac dysfunction and fibrosis in an isoproterenol-induced heart failure model, in part through suppression of MMP12 expression [29]. Given the established involvement of MMPs in vascular remodeling and the recognized pro-calcific role of oxidative stress, we hypothesized that a specific MMP program may couple ECM remodeling to NOX2-associated redox signaling and osteogenic reprogramming under calcifying conditions. Here, we tested this hypothesis using YAK577 in CM-treated VSMCs and a vitamin D_3_-induced mouse model of arterial calcification, combined with MMP14 loss- and gain-of-function approaches. Our findings suggest that YAK577 attenuates calcification and osteogenic marker induction in association with reduced MMP14 expression and diminished NOX2-derived ROS signaling, supporting an HDAC-sensitive MMP14–NOX2/ROS axis as a potential mechanism.

## 2. Materials and Methods

### 2.1. Reagents and Plasmids

GSK2795039, a selective NOX2 inhibitor, was purchased from Sigma-Aldrich (St. Louis, MO, USA; cat. no. SML2770). Antibodies against MT-MMP1/MMP14 (sc-377097), BMP2 (sc-137087), MSX2 (sc-365232), RUNX2 (sc-390351), β-actin (sc-47778), and GFP (sc-9996) were purchased from Santa Cruz Biotechnology (Dallas, TX, USA). Anti-NOX2 antibody (ab106940) was purchased from Abcam (Cambridge, UK). Anti-phospho-p47phox (Ser370) antibody (PA5-36863) was purchased from Invitrogen/Thermo Fisher Scientific (Waltham, MA, USA), and anti-p47phox antibody (F3Y6C; cat. no. 63290) was purchased from Cell Signaling Technology (Danvers, MA, USA). For Western blot analysis, primary antibodies were used at the following dilutions: RUNX2, 1:600; MSX2, 1:600; BMP2, 1:200; β-actin, 1:1000; NOX2, 1:1000; MMP14, 1:1000; phospho-p47phox, 1:500; and p47phox, 1:500. The TOPview™ Prestained Protein Ladder Marker (3.5–240 kDa; Enzynomics, Daejeon, Republic of Korea; cat. no. EOP001S) was used as the molecular weight marker for Western blot analysis.

Alexa Fluor™ 488-conjugated rabbit anti-mouse IgG (H + L) secondary antibody (cat. no. A11059), Texas Red™-X Phalloidin (cat. no. T7471), and ProLong™ Gold Antifade Mountant with DAPI (cat. no. P36931) were purchased from Invitrogen™, Thermo Fisher Scientific (Waltham, MA, USA) and used for fluorescence staining. Dihydroethidium (DHE; cat. no. D1168) was obtained from Thermo Fisher Scientific (Waltham, MA, USA). Alizarin Red S staining solution (2%; cat. no. BA054) was purchased from BioSolution (Suwon, Republic of Korea). Silver nitrate (AgNO_3_; cat. no. 209139) and N-acetyl-L-cysteine (NAC; cat. no. A9165) were purchased from Sigma-Aldrich (St. Louis, MO, USA).

Cholecalciferol (vitamin D_3_; cat. no. C9756), dimethyl sulfoxide (DMSO; cat. no. D2650), D-(+)-glucose (cat. no. G7528), and Kolliphor^®^ EL (cat. no. C5135) were purchased from Sigma-Aldrich (St. Louis, MO, USA) and used for preparation of the vitamin D_3_ solution. The novel histone deacetylase inhibitor YAK577 was synthesized in the laboratory of Prof. Kurz as previously described [29]. The plasmids used in this study included the pCMV3-C-GFPSpark control vector (CV026, Sino Biological Inc., Beijing, China) and a GFP-tagged mouse MMP14 expression plasmid, C-GFPSpark (MG51026-ACG, Sino Biological Inc., Beijing, China).

### 2.2. Cell Cultures and Cell Viability

Rat vascular smooth muscle cells (VSMCs) were isolated from the thoracic aortas of 7-week-old male Sprague–Dawley rats using an enzymatic digestion protocol based on our previously published method, with minor modifications [30]. Briefly, the thoracic aortas were excised under sterile conditions, and the endothelial layer was gently removed using a sterile cotton swab. The remaining tissue was minced into small pieces with sterile scissors and digested in a collagenase/elastase II solution at 37 °C with gentle agitation until the tissue was nearly dissociated. The resulting cell suspension was resuspended in Dulbecco’s modified Eagle’s medium (DMEM) containing 10% fetal bovine serum (FBS) and antibiotics, and cultured at 37 °C in a humidified atmosphere of 5% CO_2_. The culture medium was replaced every 2 days. VSMCs between passages 6 and 8 were used for all experiments.

For the cell viability assay, VSMCs were treated with various concentrations of YAK577 for 24 h under normal culture conditions. After treatment, MTT reagent was added and cells were incubated for 2 h at 37 °C. The medium was removed, and the formazan crystals were dissolved in dimethyl sulfoxide (DMSO). Absorbance was measured at 570 nm using a Multiskan™ FC microplate photometer (Thermo Fisher Scientific, Vantaa, Finland).

### 2.3. VSMC Calcification and Calcium Assay

To induce calcification, VSMCs at approximately 80% confluence were cultured in calcification medium (CM) consisting of Dulbecco’s modified Eagle’s medium (DMEM) supplemented with 10% fetal bovine serum (FBS), 6 mM inorganic phosphate, and 50 μg/mL ascorbic acid for 4–6 days.

For calcium quantification, cells and aortic tissues were decalcified in 0.6 N HCl at 4 °C for 24 h. Calcium content was determined using a colorimetric calcium assay kit (QuantiChrom™ Calcium Assay Kit, BioAssay Systems, Hayward, CA, USA) according to the manufacturer’s instructions.

To normalize calcium levels, cellular protein was extracted using 0.1 N NaOH containing 0.1% SDS and quantified by a protein assay. For tissue samples, calcium content was normalized to tissue weight (μg Ca^2+^ per mg tissue).

### 2.4. Histology and Calcification Staining (H&E, Alizarin Red S, and Von Kossa)

#### 2.4.1. Aortic Tissue Collection and Regional Allocation

At the experimental endpoint, mice were euthanized and the thoracic cavity was opened to expose the heart and great vessels. The aorta was carefully dissected and surrounding adipose and connective tissues were removed. Systemic perfusion was not performed prior to tissue collection. For histological analyses, including H&E, Alizarin Red S, and Von Kossa staining, the aortic arch was isolated, fixed in formaldehyde, and processed for paraffin embedding as described below. The aortic arch was also used for calcium quantification assays.

Other aortic segments were allocated for downstream biochemical analyses, including RNA extraction and protein analysis. The specific procedures and the amount of tissue used for these assays are described in the corresponding subsections of the Methods.

#### 2.4.2. Hematoxylin and Eosin (H&E) Staining

Aortic arch tissues were fixed in 3.7% formaldehyde, dehydrated, and embedded in paraffin. Paraffin-embedded tissues were sectioned at a thickness of 5 μm, deparaffinized, and rehydrated through a graded ethanol series. For H&E staining, sections were stained with hematoxylin for 4 min, rinsed under running tap water for 5 min, and counterstained with eosin for 1 min. The slides were then dehydrated, cleared in xylene, and mounted with Canada balsam for microscopic observation under a Nikon Eclipse 80i microscope (Nikon, Tokyo, Japan). Aortic wall thickness was measured on cross-sections using ImageJ software [31] (version 1.53t; National Institutes of Health, Bethesda, MD, USA) by determining the wall thickness at 8 different regions per section, and the mean value was used for analysis.

#### 2.4.3. Alizarin Red S Staining

For cultured VSMCs, after two washes with phosphate-buffered saline (PBS), cells were fixed with 1.85% paraformaldehyde for 15 min at room temperature and stained with 2% Alizarin Red S solution (pH 4.2) for 15 min until a distinct red-orange coloration was observed. The cells were then rinsed thoroughly deionized water to remove excess dye.

For aortic arch sections, paraffin-embedded sections (5 μm) were deparaffinized, rehydrated, and briefly rinsed with distilled water. The sections were incubated with 2% Alizarin Red S solution (pH 4.2) for approximately 1 min until red-orange calcium deposits were visible under a light microscope. Excess dye was gently removed, and the slides were dehydrated in acetone, cleared sequentially with an acetone–xylene mixture and pure xylene, and mounted for observation under a Nikon Eclipse 80i microscope (Nikon, Tokyo, Japan).

#### 2.4.4. Von Kossa Staining

For cultured VSMCs, after two washes with PBS, cells were fixed with 1.85% formaldehyde for 15 min at room temperature. Following two additional PBS washes, the cells were incubated with 5% silver nitrate solution and exposed to ultraviolet light for 60 min to visualize calcium phosphate deposits as black or dark brown metallic silver. Unreacted silver nitrate was removed by rinsing the cells twice with deionized water, and images were captured using a Nikon Eclipse 80i microscope (Nikon, Tokyo, Japan) for documentation.

For aortic arch sections, deparaffinized and rehydrated sections were rinsed thoroughly with distilled water and incubated in 5% silver nitrate solution. The slides were exposed to ultraviolet light for 60 min until calcium deposits appeared black. Residual silver nitrate was removed by washing with distilled water, and the sections were counterstained with eosin for 1 min, rinsed, dehydrated through graded ethanol, cleared in xylene, and mounted for microscopic evaluation under a Nikon Eclipse 80i microscope (Nikon, Tokyo, Japan).

### 2.5. Immunofluorescence Staining of GFP-MMP14-Transfected A10 Cells

The rat A10 vascular smooth muscle cell line (ATCC CRL-1476) was obtained from the American Type Culture Collection (ATCC, Manassas, VA, USA). A10 cells were transfected with either a pCMV3-C-GFPSpark control vector or a GFP-tagged mouse MMP14 plasmid. Two days after transfection, the cells were washed twice with phosphate-buffered saline (PBS, 1×) and fixed with 1.85% paraformaldehyde for 15 min at room temperature. After two additional PBS washes, the cells were permeabilized with 0.1% Triton X-100 for 10 min and washed twice with PBS.

Cells were then blocked with 3% normal goat serum for 60 min at room temperature and incubated overnight at 4 °C with an anti-GFP primary antibody (1:500 dilution). After three PBS washes, the cells were incubated with goat-anti-mouse Alexa Fluor™ 488-conjugated secondary antibody to enhance GFP-MMP14 signals for 45 min at room temperature in the dark. F-actin was stained using Texas Red™-X Phalloidin.

Following three final PBS washes, the coverslips were gently removed, blotted to eliminate excess liquid, and mounted with DAPI-containing mounting medium. Fluorescent images were captured using a fluorescence microscope (Eclipse 80i, Nikon, Tokyo, Japan).

### 2.6. DHE Staining for Intracellular ROS

Intracellular ROS generation was assessed using the superoxide-sensitive fluorescent probe dihydroethidium (DHE). VSMCs were cultured in growth medium (GM) or calcification medium for 4 days to induce calcification. For ROS measurements, cells were treated with YAK577 (1 μM) or vehicle for 24 h, or with N-acetylcysteine (NAC, 1 mM) or vehicle for 9 h.

For the NOX2 inhibition experiment, VSMCs were seeded and allowed to attach overnight. On the following day, cells were pretreated with GSK2795039, a NOX2 inhibitor, at 20 μM or DMSO vehicle for 1 h. The medium was then replaced with GM or CM containing GSK2795039 or DMSO vehicle, and cells were further cultured for 24 h before DHE staining.

After treatment, cells were gently washed twice with pre-warmed PBS and incubated with DHE working solution (5 μM in serum-free medium) for 15 min at 37 °C in the dark. The DHE-containing medium was removed, and cells were rinsed with PBS. Cells were then mounted using a DAPI-containing mounting medium to counterstain nuclei. For the YAK577 and NAC experiments, DHE fluorescence was detected using a TRITC filter set/channel (EX 540/25 nm, DM565, BA605/55 nm) on a Nikon Eclipse 80i fluorescence microscope (Nikon, Tokyo, Japan). For the GSK2795039 experiment, fluorescence images were acquired using a laser scanning confocal microscope (LSM510, Carl Zeiss, Oberkochen, Germany), with DHE fluorescence captured in the red channel and DAPI nuclear staining captured in the blue channel. All images within the same experiment were acquired under identical exposure and imaging settings. Fluorescence intensity was quantified using ImageJ software [31] (version 1.53t; National Institutes of Health, Bethesda, MD, USA) by measuring the mean DHE fluorescence intensity per field and normalizing to the corresponding control group.

### 2.7. Quantitative Reverse Transcription Polymerase Chain Reaction (qRT-PCR)

Total RNA was isolated from cultured cells or aortic tissues using TRIzol™ reagent (Invitrogen, Carlsbad, CA, USA) according to the manufacturer’s protocol. RNA concentration and purity were determined by measuring absorbance at 260 nm using a spectrophotometer (ACTGene ASP-2680, Piscataway, NJ, USA). Complementary DNA (cDNA) was synthesized from total RNA using TOPscript™ RT DryMIX (Enzynomics, Daejeon, Republic of Korea).

Quantitative real-time PCR (qRT-PCR) was performed using a SYBR^®^ Green PCR Master Mix (Applied Biosystems, Foster City, CA, USA) and gene-specific primers. Relative mRNA expression levels were calculated using the 2^−ΔΔCt^ method, with Gapdh or β-actin serving as the internal control. The primer sequences used for qRT-PCR are listed in Table 1.

### 2.8. Western Blotting

Total protein was extracted from cultured cells or aortic tissues using RIPA lysis buffer as previously described [29]. For aortic protein extraction, frozen thoracic and abdominal aortas were minced on ice, lysed in RIPA buffer, and homogenized on ice. The lysates were centrifuged at 13,000 rpm for 20 min at 4 °C, and the supernatants were collected. Protein concentration was determined using a bicinchoninic acid (BCA) protein assay kit (Pierce, Rockford, IL, USA). Equal amounts of protein (30 μg per lane) were separated by sodium dodecyl sulfate–polyacrylamide gel electrophoresis (SDS–PAGE) and transferred onto polyvinylidene fluoride (PVDF) membranes (Millipore, Billerica, MA, USA). After transfer, the prestained marker bands were clearly visible on the PVDF membrane, and their positions and corresponding molecular weights were manually marked with a ballpoint pen to facilitate identification of target protein bands during subsequent analysis.

The membranes were blocked with 5% skim milk in Tris-buffered saline containing 0.1% Tween-20 (TBST) for 1 h at room temperature and then incubated overnight at 4 °C with primary antibodies (1:1000 dilution). After three washes with TBST, the membranes were incubated with horseradish peroxidase (HRP)-conjugated anti-mouse IgG secondary antibody (1:5000; Cell Signaling Technology, Danvers, MA, USA) for 1 h at room temperature.

Protein bands were visualized using enhanced chemiluminescence (ECL) substrate (Millipore, Billerica, MA, USA) and imaged using an iBright FL1000 imaging system (Thermo Fisher Scientific, Waltham, MA, USA). Because prestained molecular weight markers are not HRP-conjugated, the chemiluminescent signal and the visual marker bands were captured separately and merged by the imaging software to determine band positions and molecular weights. β-actin was used as the internal loading control for normalization.

### 2.9. Knockdown of MMP14

For gene silencing, VSMCs were transfected with either control or MMP14 small interfering RNA (siRNA; 100 nM; Bioneer, Daejeon, Republic of Korea) using Lipofectamine™ RNAiMAX reagent (Invitrogen, Carlsbad, CA, USA) according to the manufacturer’s instructions. Cells were incubated for 48 h after transfection, and the efficiency of MMP14 knockdown was verified by quantitative reverse transcription PCR (qRT-PCR).

### 2.10. Overexpression of MMP14

For overexpression experiments, A10 cells were transfected with 1 μg of either empty vector or GFP–MMP14 construct using the PLUS™ reagent and Lipofectamine™ reagent (Invitrogen, Carlsbad, CA, USA) at a 6:2 ratio, according to the manufacturer’s instructions. The cells were cultured for 48 h after transfection. The efficiency of MMP14 overexpression was confirmed by quantitative reverse transcription PCR (qRT-PCR), Western blotting, and GFP fluorescence staining.

### 2.11. Vascular Calcification in Mice

All animal experiments were approved by the Institutional Animal Care and Use Committee of Chonnam National University Medical School (CNUH IACUC-22023; approved on 26 September 2022) and were conducted in accordance with the ARRIVE guidelines 2.0 and the Guide for the Care and Use of Laboratory Animals (8th edition, National Research Council, Washington, DC, USA, 2011 [32]). Male C57BL/6N mice (8 weeks old, 21–24 g) were housed under controlled conditions (22 ± 2 °C, 50–60% humidity, 12 h light/dark cycle) with free access to food and water. Mice were randomly assigned to experimental groups, and investigators performing histological and molecular analyses were blinded to group allocation. Humane endpoints were predefined, and animals showing signs of severe distress were euthanized according to institutional guidelines. Male C57BL/6N mice were randomly assigned to three groups: control (*n* = 14), vitamin D_3_-treated (*n* = 12), and vitamin D_3_+ YAK577-treated (*n* = 12).

To induce vascular calcification, cholecalciferol was first dissolved in DMSO, followed by the addition of Kolliphor^®^ EL as a solubilizing agent. In a separate tube, glucose was dissolved in sterile distilled water and then added to the cholecalciferol mixture. The final solution was thoroughly vortexed and shaken for 15 min before use [33]. The corresponding vehicle was prepared using the same formulation without cholecalciferol. Mice were subcutaneously injected with vitamin D_3_ (120 µL; 4 × 10^5^ IU/kg/day) for five consecutive days. This dosing regimen was selected as a high-dose vitamin D_3_ overload protocol widely used to rapidly and reproducibly induce medial vascular calcification in mice for mechanistic studies, rather than representing physiological or nutritional vitamin D_3_ exposure [34,35]. YAK577 (10 mg/kg/day) or vehicle was administered intraperitoneally from day 2 to day 8 following the first vitamin D_3_ injection. Body weight was recorded every other day to monitor general health. Aortic tissues were collected on day 9.

Mice were euthanized by CO_2_ inhalation, and the entire aorta, including the aortic arch, thoracic aorta, and abdominal aorta, was dissected and immediately placed in ice-cold 1× PBS. Surrounding adipose and connective tissues were carefully removed. For calcium quantification, the aortic arch was incubated in 0.6 N HCl at 4 °C for 24 h. For RNA and protein analyses, the thoracic and abdominal aortas were snap-frozen in liquid nitrogen and stored at −80 °C until use. For histological analysis, separate aortic samples were collected in the same manner, fixed in neutral buffered formalin for 24 h, and then embedded in paraffin. Sections of the aortic arch were used for H&E, Alizarin Red S, and Von Kossa staining.

### 2.12. Statistical Analysis

All statistical analyses were performed using GraphPad Prism version 8.0 (GraphPad Software, San Diego, CA, USA). Data are expressed as the mean ± standard error of the mean (SEM). Normality of data distribution was assessed using the Shapiro–Wilk test prior to parametric analysis, and variance homogeneity was evaluated. If assumptions were not met, appropriate non-parametric tests were applied. Differences among three or more groups were analyzed by one-way analysis of variance (ANOVA) followed by Bonferroni’s post hoc test. A *p* value < 0.05 was considered statistically significant.

### 2.13. Generative AI Statement

Generative AI statement: Generative artificial intelligence (GenAI) tools were not used to generate scientific content (including study design, data collection, data analysis, data interpretation, figures/graphics, or numerical results) in this manuscript. Any language editing was limited to superficial improvements (grammar, spelling, punctuation, and formatting).

## 3. Results

### 3.1. The Novel HDAC Inhibitor YAK577 Reduces Calcium Deposition in Calcified VSMCs

**Figure 1:**
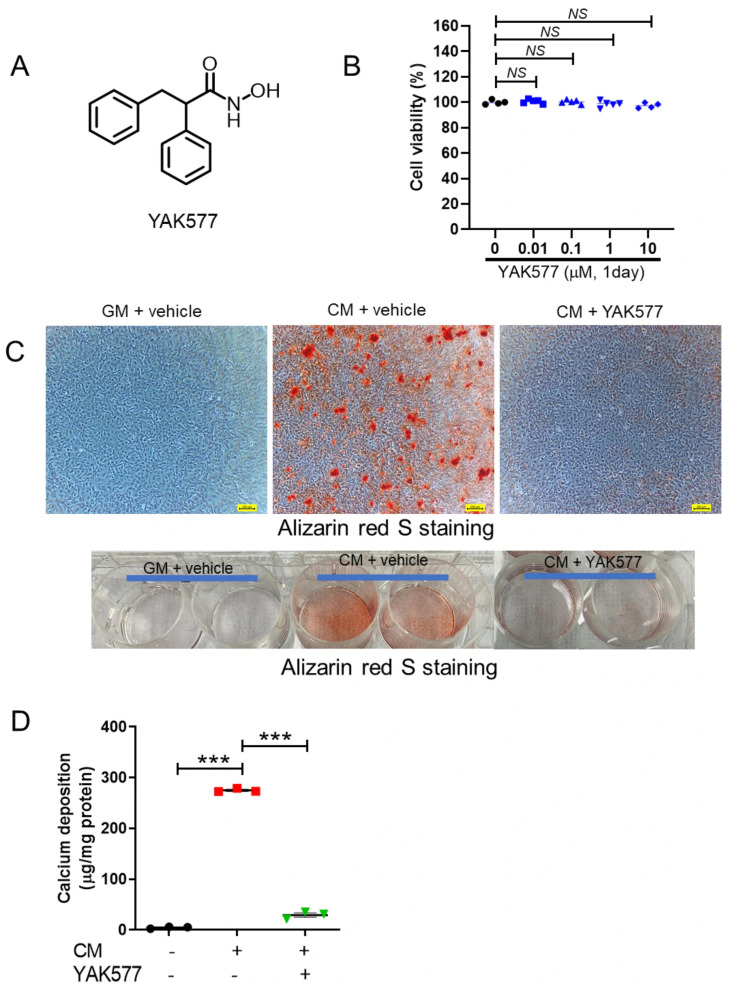
The HDAC inhibitor YAK577 reduces CM-induced vascular calcification in VSMCs. (**A**) Chemical structure of YAK577. (**B**) Effect of YAK577 on VSMC viability determined by MTT assay (*n* = 4, 5, 5, 5, 4). (**C**) Representative images of Alizarin Red S-stained VSMCs observed under a light microscope. Scale bar = 200 μm. Bottom: Macroscopic photograph of Alizarin Red S-stained VSMCs captured using a smartphone camera, showing red coloration corresponding to calcium deposition. (**D**) Quantitative analysis of calcium content in CM-treated VSMCs with or without YAK577 treatment (*n* = 3, 3, 3). Data are presented as mean ± SEM. *** *p* < 0.001; NS, not significant. GM, growth medium; CM, calcification medium. Each dot represents one independent biological experiment (not a technical replicate).

To investigate how the novel HDAC inhibitor YAK577 [29] influences this process, we first assessed its cytotoxicity. YAK577, a hydroxamic acid-based compound (Figure 1A), showed no cytotoxic effects on vascular smooth muscle cells (VSMCs) up to a concentration of 10 μM, as determined by MTT assay (Figure 1B).

VSMC calcification was induced by culturing cells in calcification medium (CM) containing inorganic phosphate and ascorbic acid for 4 days, with YAK577 treatment administered 1 day before cell harvesting. Alizarin Red S staining revealed substantial calcium deposition in CM-treated VSMCs compared with the growth medium (GM) control, whereas YAK577 markedly reduced calcium accumulation (Figure 1C). Quantitative analysis using a calcium assay kit confirmed that YAK577 significantly attenuated the CM-induced increase in calcium content (Figure 1D).

### 3.2. YAK577 Suppresses CM-Induced Oxidative Stress by Inhibiting NOX2/p47phox Activation in VSMCs

**Figure 2:**
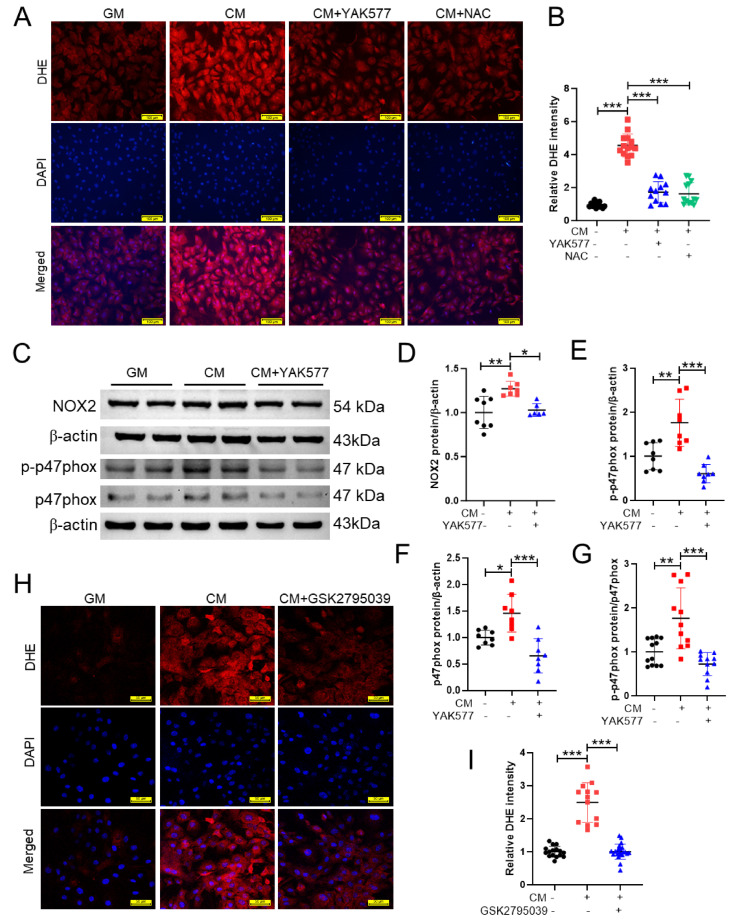
YAK577 suppresses CM-induced oxidative stress through inhibition of the NOX2/p47phox axis in VSMCs. (**A**) Representative dihydroethidium (DHE) fluorescence images showing intracellular ROS levels in VSMCs cultured in GM, CM, CM + YAK577, or CM + NAC. Scale bar = 100 μm. (**B**) Quantification of DHE fluorescence intensity in the indicated groups (*n* = 14, 14, 12, 13). (**C**–**G**) Representative Western blot images and corresponding densitometric analyses of NOX2, phosphorylated p47phox at Ser370, and total p47phox in VSMCs cultured in GM, CM, or CM + YAK577. NOX2 (*n* = 8, 7, 6), phosphorylated p47phox (*n* = 8, 8, 8), and total p47phox (*n* = 8, 8, 8) were normalized to β-actin, and the phosphorylated p47phox/total p47phox ratio (*n* = 12, 11, 11) was calculated to assess p47phox activation. (**H**) Representative DHE fluorescence images of VSMCs treated with DMSO vehicle or GSK2795039 and subsequently cultured in GM or CM. Scale bar = 50 μm. (**I**) Quantification of DHE fluorescence intensity in the indicated groups (*n* = 16, 13, 22). Data are presented as mean ± SEM. *** *p* < 0.001; ** *p* < 0.01; * *p* < 0.05. GM, growth medium; CM, calcification medium; NAC, N-acetylcysteine. For DHE quantification, each dot represents one quantified microscopic field. For Western blot quantification, each dot represents one independent biological experiment (not a technical replicate).

Because oxidative stress is a key driver of osteogenic reprogramming and vascular calcification, we next examined whether YAK577 modulates intracellular ROS in calcified VSMCs. Dihydroethidium (DHE) staining demonstrated that CM markedly increased ROS levels compared with GM, whereas YAK577 substantially reduced CM-induced ROS accumulation (Figure 2A,B). Treatment with the ROS scavenger N-acetylcysteine (NAC) similarly decreased CM-induced ROS, supporting a pivotal role of oxidative stress in this model (Figure 2A,B).

To further explore the potential source of ROS production, we examined the NOX2/p47phox axis, a major NADPH oxidase-dependent ROS-generating pathway. Western blot analysis showed that CM significantly increased NOX2 protein expression, whereas YAK577 markedly suppressed this elevation (Figure 2C,D). In parallel, CM enhanced phosphorylation of p47phox at Ser370 as well as total p47phox protein expression, both of which were reduced by YAK577 treatment (Figure 2C,E,F). Importantly, the ratio of phosphorylated p47phox to total p47phox was also increased under CM stimulation and significantly decreased by YAK577 (Figure 2G), suggesting that CM not only upregulates p47phox expression but also promotes p47phox activation, while YAK577 inhibits both processes.

To functionally verify the contribution of NOX2 activity to CM-induced ROS generation, VSMCs were treated with the NOX2 inhibitor GSK2795039 before exposure to GM or CM. GSK2795039 markedly attenuated CM-induced DHE fluorescence compared with CM alone (Figure 2H,I), indicating that NOX2 activity is involved in CM-triggered ROS accumulation in VSMCs. Together, these findings support the notion that YAK577 alleviates CM-induced oxidative stress, at least in part, by suppressing NOX2 expression and p47phox activation.

### 3.3. YAK577 Attenuates the Expression of Pro-Calcification Marker Genes in Calcified VSMCs

**Figure 3:**
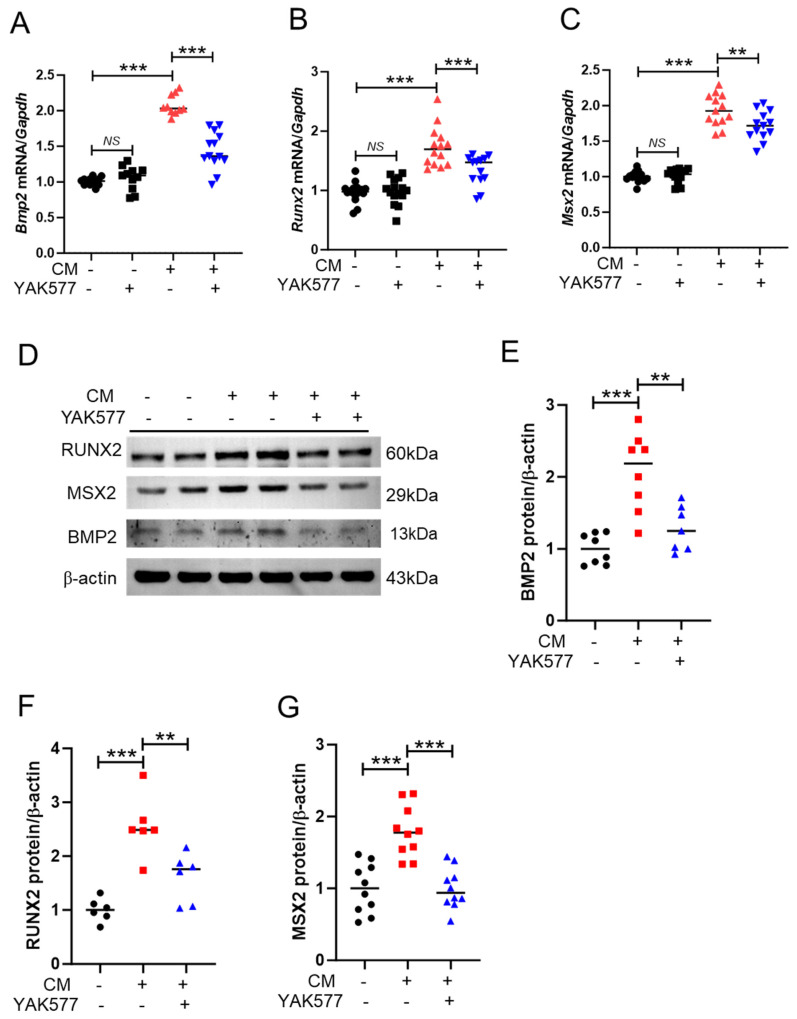
YAK577 attenuates the expression of pro-calcification marker genes in vitro. (**A**–**C**) qRT-PCR analysis of *Bmp2* (*n* = 14, 11, 10, 13), *Runx2* (*n* = 9, 7, 8, 8), and *Msx2* (*n* = 14, 14, 13, 13) mRNA levels in VSMCs cultured in calcification medium (CM) with or without YAK577 treatment. (**D**–**G**) Representative Western blots and corresponding densitometric quantification of BMP2 (*n* = 8, 8, 7), RUNX2 (*n* = 6, 6, 6), and MSX2 (*n* = 10, 10, 10) protein expression. β-actin was used as a loading control. Data are presented as mean ± SEM. *** *p* < 0.001; ** *p* < 0.01; NS, not significant. GM, growth medium; CM, calcification medium. Each dot represents one independent biological experiment (not a technical replicate).

To determine whether YAK577 suppresses osteogenic reprogramming during calcification, we quantified the expression of pro-calcification markers. CM treatment significantly upregulated *Bmp2*, *Runx2*, and *Msx2* mRNA levels, whereas YAK577 markedly reduced the CM-induced increases (Figure 3A–C). Consistent with the transcriptional changes, Western blot analysis confirmed that YAK577 attenuated CM-induced upregulation of BMP2, RUNX2, and MSX2 proteins (Figure 3D–G). These findings suggest that YAK577 suppresses the osteogenic program associated with VSMC calcification.

### 3.4. YAK577 Selectively Reduces MMP14 Expression in Calcified VSMCs

**Figure 4:**
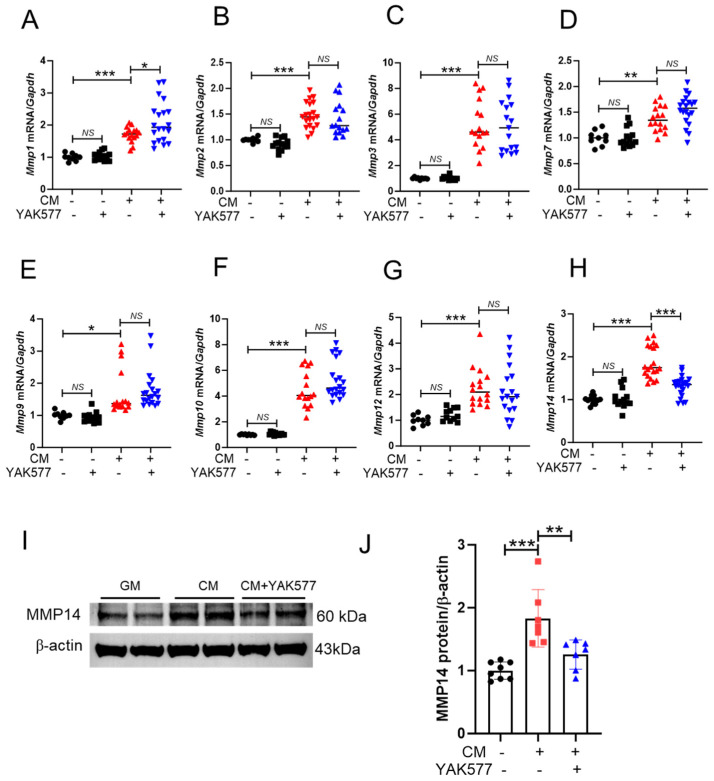
YAK577 preferentially suppresses MMP14 expression among MMP family members in calcified VSMCs. (**A**–**H**) qRT-PCR analysis of *Mmp1* (*n* = 9, 13, 16, 20), *Mmp2* (*n* = 9, 13, 16, 20), *Mmp3* (*n* = 9, 11, 16, 17), *Mmp7* (*n* = 9, 13, 16, 20), *Mmp9* (*n* = 9, 13, 16, 20), *Mmp10* (*n* = 9, 13, 16, 20), *Mmp12* (*n* = 9, 11, 16, 18), and *Mmp14* (*n* = 13, 13, 20, 23) mRNA levels in VSMCs cultured in calcification medium (CM) with or without YAK577 treatment. mRNA levels were normalized to *Gapdh* expression. (**I**,**J**) Representative Western blots and corresponding densitometric quantification showing MMP14 protein expression in VSMCs treated with CM in the presence or absence of YAK577 (*n* = 8, 7, 7). Data are presented as mean ± SEM. *** *p* < 0.001; ** *p* < 0.01; * *p* < 0.05; NS, not significant. GM, growth medium; CM, calcification medium. Each dot represents one independent biological experiment (not a technical replicate).

Matrix metalloproteinases (MMPs) have been implicated in the progression of vascular calcification [15,36]. To determine whether YAK577 regulates MMP expression, mRNA levels of several MMP family members were analyzed by qRT-PCR. The expression of multiple MMPs was elevated in VSMCs cultured in calcification medium (CM); however, only *Mmp14* expression was significantly reduced by YAK577 treatment (Figure 4A–H). Consistently, Western blot analysis confirmed that YAK577 markedly attenuated CM-induced upregulation of MMP14 protein expression (Figure 4I,J).

### 3.5. MMP14 Knockdown Attenuates Vascular Calcification in VSMCs

**Figure 5:**
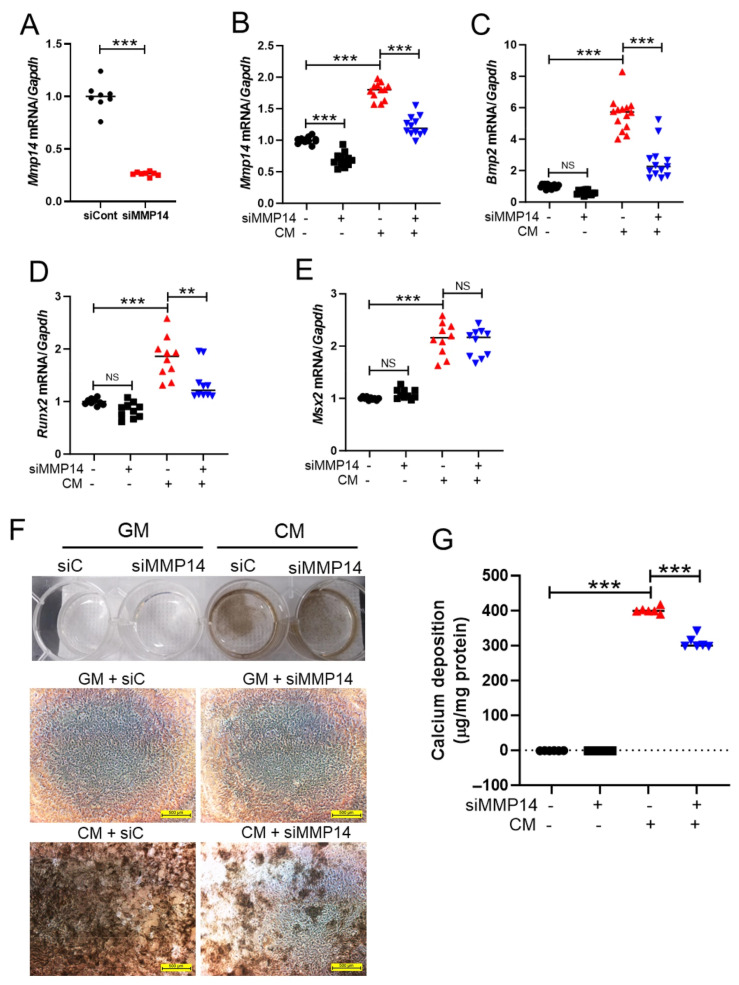
Knockdown of MMP14 reduces vascular calcification and osteogenic marker expression in VSMCs. (**A**–**E**) VSMCs were transfected with siMMP14 and cultured in calcification medium (CM). mRNA levels of *Mmp14* (12, 12, 12, 12), *Bmp2* (14, 14, 14, 13), *Runx2* (10, 10, 10, 10), and *Msx2* (10, 10, 10, 10) were analyzed by qRT-PCR. (**F**) Representative von Kossa staining images showing calcium deposition in CM-treated VSMCs after siMMP14 transfection. Scale bar = 500 μm. (**G**) Quantitative analysis of calcium content demonstrating that MMP14 knockdown significantly reduces CM-induced calcification in VSMCs (*n* = 6, 6, 6, 6). Data are presented as mean ± SEM. *** *p* < 0.001; ** *p* < 0.01; NS, not significant. GM, growth medium; CM, calcification medium. Each dot represents one independent biological experiment (not a technical replicate).

To further elucidate the role of MMP14 in vascular calcification, VSMCs were transfected with small interfering RNA targeting MMP14 (siMMP14). Knockdown of MMP14 significantly reduced CM-induced upregulation of *Mmp14*, *Runx2*, and *Bmp2* mRNA expression, whereas *Msx2* levels remained largely unchanged (Figure 5A–E).

Von Kossa staining showed a marked increase in calcium deposition in VSMCs treated with siC + CM compared with those treated with siC + GM (control). Notably, this increase in calcium accumulation was significantly attenuated by siMMP14 treatment (Figure 5F). Consistently, quantitative calcium assays confirmed that siMMP14 markedly suppressed CM-induced calcium deposition in VSMCs (Figure 5G).

### 3.6. MMP14 Overexpression Promotes NOX2 Upregulation and Enhances Osteogenic Marker Expression in A10 Cells

**Figure 6:**
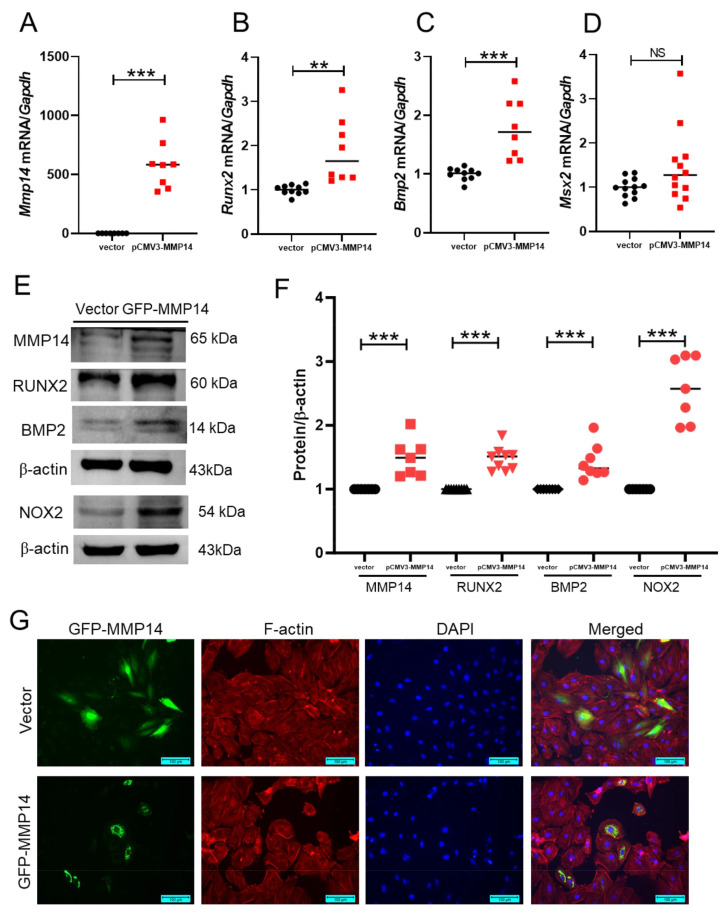
MMP14 overexpression promotes NOX2 upregulation and enhances osteogenic marker expression in A10 cells. (**A**–**D**) qRT-PCR analysis of *Mmp14* (*n* = 8, 8), *Bmp2* (*n* = 10, 8), *Runx2* (*n* = 10, 8), and *Msx2* (*n* = 12, 12) mRNA levels in A10 cells following MMP14 plasmid transfection. (**E**,**F**) Representative Western blots and corresponding densitometric quantification showing increased expression of MMP14 (*n* = 7, 7), RUNX2 (*n* = 9, 9), BMP2 (*n* = 8, 8) and NOX2 (*n* = 7, 7) proteins after MMP14 overexpression. β-actin was used as a loading control. (**G**) Representative immunofluorescence images of A10 cells transfected with vector or GFP-MMP14. GFP-MMP14 was detected with anti-GFP antibody/Alexa Fluor 488 (green), F-actin with Texas Red-X Phalloidin (red), and nuclei with DAPI (blue). Scale bar = 100 μm. Data are presented as mean ± SEM. *** *p* < 0.001; ** *p* < 0.01; NS, not significant. Each dot represents one independent biological experiment (not a technical replicate).

To further determine whether MMP14 is sufficient to drive pro-calcific signaling, we overexpressed MMP14 in A10 cells. MMP14 overexpression significantly increased the mRNA levels of calcification-related genes, including *Bmp2* and *Runx2*, while *Msx2* remained largely unchanged (Figure 6A–D). Western blot analysis confirmed elevated protein expression of MMP14, RUNX2, and BMP2 in MMP14-overexpressing cells. Importantly, NOX2 protein expression was also increased following MMP14 overexpression (Figure 6E,F), suggesting a potential link between MMP14 and oxidative stress-associated signaling. Fluorescence imaging showed strong anti-GFP immunofluorescence signals (green) in GFP-MMP14-transfected A10 cells, predominantly distributed in the perinuclear region, indicating successful transfection and intracellular localization of GFP-MMP14. F-actin was stained with Texas Red-X Phalloidin (red), and nuclei were counterstained with DAPI (blue) (Figure 6G).

### 3.7. YAK577 Attenuates Arterial Calcification and Osteogenic Reprogramming in Vitamin D_3_-Injected Mice

**Figure 7:**
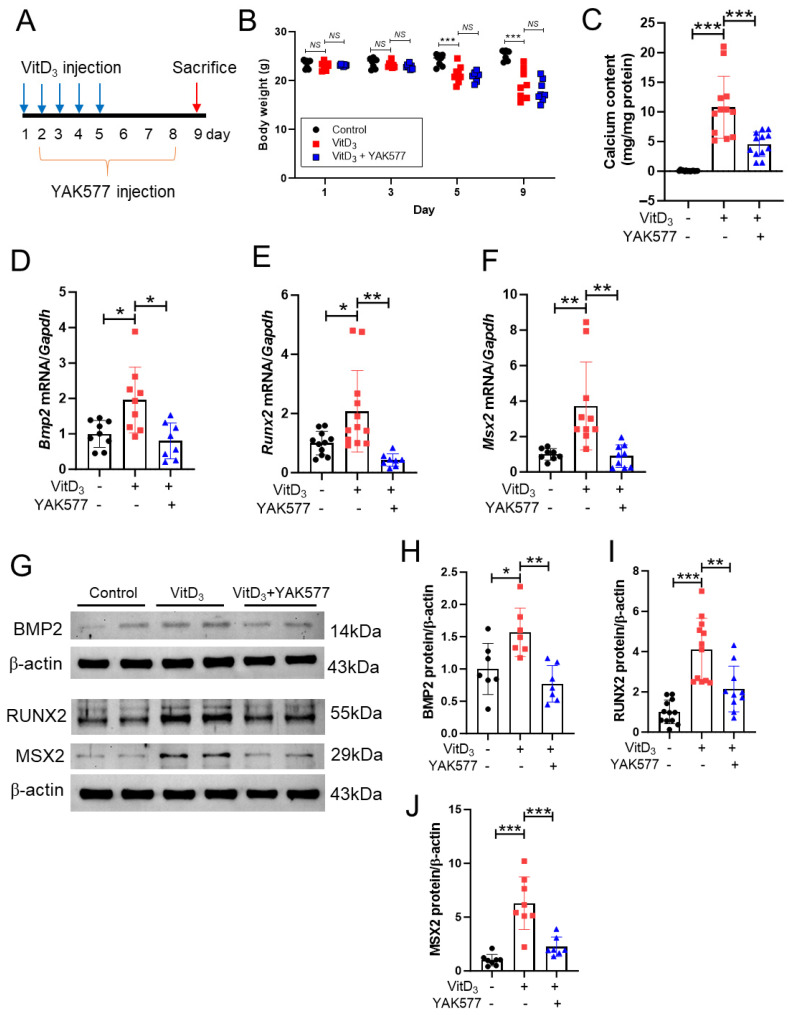
YAK577 attenuates arterial calcification and osteogenic reprogramming in vitamin D_3_-treated mice. Exact sample sizes for each analysis are indicated in the panels. Groups are control, vitamin D_3_, and vitamin D_3_ + YAK577 unless otherwise indicated. (**A**) Schematic diagram showing the experimental design of the vitamin D_3_-induced vascular calcification model and YAK577 treatment schedule. (**B**) Changes in body weight over time following vitamin D_3_ injection and YAK577 administration. (**C**) Quantitative analysis of aortic calcium content in vitamin D_3_-injected mice with or without YAK577 treatment (*n* = 14, 12, 12). (**D**–**F**) qRT-PCR analysis of *Bmp2* (*n* = 9, 9, 8), *Runx2* (*n* = 11, 12, 8), and *Msx2* (*n* = 8, 10, 9) mRNA levels in aortic tissue from vitamin D_3_-treated mice in the presence or absence of YAK577. (**G**–**J**) Representative Western blots and corresponding densitometric quantification showing the expression of MSX2 (*n* = 8, 8, 7), RUNX2 (*n* = 12, 12, 10), and BMP2 (*n* = 7, 7, 7) proteins in the aortas of vitamin D_3_-treated mice with or without YAK577. β-actin was used as a loading control. Data are presented as mean ± SEM. *** *p* < 0.001; ** *p* < 0.01; * *p* < 0.05; NS, not significant. Each dot represents one independent biological experiment (not a technical replicate).

To determine whether the anti-calcific effects of YAK577 observed in vitro could be reproduced in vivo, vascular calcification was induced in mice by subcutaneous injection of vitamin D_3_ for 5 consecutive days. YAK577 was administered intraperitoneally for 7 days, beginning on day 2 after vitamin D_3_ injection (Figure 7A). From day 5 onward, body weight was significantly decreased in vitamin D_3_-treated mice compared with the control group and was not restored by YAK577 administration (Figure 7B). Quantitative calcium assays revealed that YAK577 treatment markedly reduced aortic calcium accumulation induced by vitamin D_3_ injection (Figure 7C).

Osteogenic marker genes, including *Bmp2*, *Runx2*, and *Msx2*, were quantified by qRT-PCR. The expression of all three genes was significantly elevated in the aortas of vitamin D_3_-treated mice compared with controls and was effectively suppressed by YAK577 administration (Figure 7D–F). Consistently, Western blot analysis demonstrated that the protein expression of BMP2, RUNX2, and MSX2 was upregulated in vitamin D_3_-treated aortas, and this increase was attenuated by YAK577 treatment (Figure 7G–J).

### 3.8. YAK577 Suppresses MMP14/NOX2 Signaling and Mitigates Vitamin D_3_-Induced Vascular Injury and Calcification In Vivo

**Figure 8:**
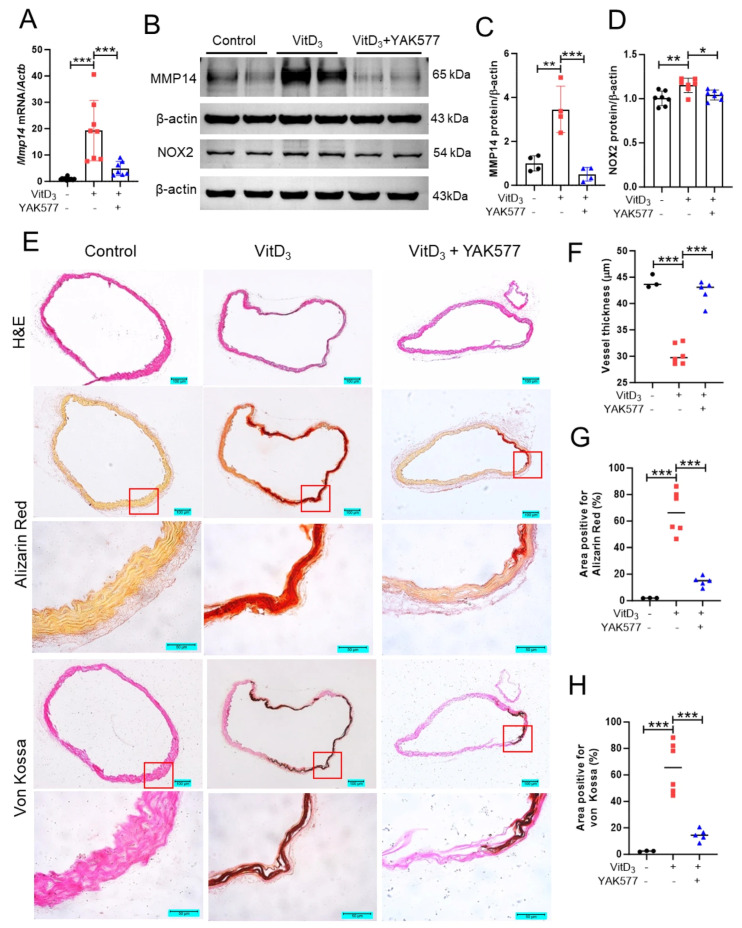
YAK577 suppresses MMP14/NOX2 signaling and mitigates vitamin D_3_-induced vascular calcification in vivo. (**A**) Representative qRT-PCR analysis with corresponding quantification showing *Mmp14* mRNA in aortic tissues from vitamin D_3_-treated mice with or without YAK577 administration (*n* = 11, 8, 7). (**B**–**D**) Representative Western blots and densitometric quantification showing MMP14 (*n* = 4, 4, 4) and NOX2 (*n* = 7, 7, 7) protein expression in the indicated groups. β-actin was used as a loading control. (**E**–**H**) Representative images and quantitative analysis of hematoxylin and eosin (H&E), Alizarin Red S, and von Kossa staining of aortic sections from control (*n* = 3), vitamin D_3_-treated (*n* = 6), and vitamin D_3_ + YAK577-treated mice (*n* = 5). Scale bars: For H&E staining, 100 μm. For Alizarin Red S and Von Kossa staining, the upper panels show 100 μm and the lower panels show 50 μm. Data are presented as mean ± SEM. *** *p* < 0.001; ** *p* < 0.01; * *p* < 0.05. Each dot represents one independent biological experiment (not a technical replicate).

Given that YAK577 selectively downregulated MMP14 in vitro, we next examined whether this regulation also occurs in vivo. In the vitamin D_3_ model, *Mmp14* mRNA and MMP14 protein levels were markedly elevated in aortic tissues, and YAK577 significantly attenuated these increases (Figure 8A–C). Notably, NOX2 protein expression was also upregulated in vitamin D_3_-treated aortas and was reduced by YAK577 administration (Figure 8D), supporting the notion that YAK577 inhibits oxidative stress-associated signaling in vivo. Histological analyses of aortic arch sections further corroborated the protective effect of YAK577. H&E staining showed medial layer disruption and structural damage in vitamin D_3_-treated mice, which was markedly alleviated by YAK577. In parallel, von Kossa and Alizarin Red S staining revealed extensive calcium deposition in the aortic media after vitamin D_3_ treatment, whereas YAK577 markedly reduced mineral accumulation. Quantification of H&E, von Kossa, and Alizarin Red S staining confirmed the protective effects of YAK577 on vascular structure and calcification burden (Figure 8E–H). In addition, several other MMP family members assessed in aortic tissues were elevated in the vitamin D_3_ model and were reduced by YAK577 (Supplementary Figure S1A–G).

## 4. Discussion

This study provides evidence that the novel hydroxamic acid-based HDAC inhibitor YAK577 attenuates vascular calcification in CM-treated VSMCs and in a vitamin D_3_-induced mouse model. Across both systems, YAK577 reduced mineral deposition and suppressed osteogenic markers (BMP2, RUNX2, and MSX2), and in vivo it improved calcification-associated histological changes and medial integrity. In parallel, our redox readouts—highly relevant to Antioxidants—showed that calcifying stimuli increased intracellular ROS and NOX2 expression, whereas YAK577 decreased ROS accumulation and downregulated NOX2. Taken together, these findings are consistent with our working hypothesis that epigenetic modulation can restrain calcification-associated phenotypic switching of VSMCs and that redox imbalance is an integral component of this process.

Our data also help frame the ongoing controversy regarding HDAC inhibition and vascular calcification. Prior reports have described bidirectional effects, with some pan-HDAC inhibitors promoting calcification and other studies indicating context- and target-dependent outcomes [26,27]. In addition, evidence from atherosclerosis-relevant models supports a role for class IIa HDACs in calcification and VSMC phenotype regulation [28]. In the present study, YAK577 exhibited reproducible anti-calcific effects in vitro and in vivo at non-cytotoxic concentrations, supporting the interpretation that it modulates calcification-relevant signaling rather than causing nonspecific loss of cell viability. Although we did not define HDAC isoform engagement here, the consistency across models suggests that YAK577 influences an epigenetically regulated network that intersects with matrix remodeling and NOX-dependent redox signaling [23,37].

Mechanistically, the most parsimonious interpretation of our findings supports an HDAC-sensitive pathway that converges on MMP14 and is associated with NOX2-derived ROS during calcification. Among several MMPs induced under calcifying conditions, YAK577 preferentially reduced MMP14 expression, and this pattern was recapitulated in vitamin D_3_-treated aortas. Functional perturbation strengthened the biological relevance of MMP14: MMP14 knockdown attenuated mineral deposition and reduced osteogenic gene induction, whereas MMP14 overexpression enhanced osteogenic signaling. However, the partial reduction in calcification observed with MMP14 knockdown indicates that MMP14 likely represents one contributing mechanism rather than the sole determinant of the anti-calcific effects observed with YAK577. These observations align with established roles of MMP14 as a membrane-type metalloproteinase that governs pericellular matrix remodeling and protease cascades, including pro-MMP2 activation [21], and with prior links between MMP activity and vascular calcification [38,39,40,41]. In addition, histopathological evidence has shown that MMP14 immunoreactivity can be detected in unicommissural aortic valves, including staining adjacent to and within calcific nodules, supporting an association between MMP14 and calcified lesions, although the underlying mechanism remains incompletely understood [42].

It is also noteworthy that in our previous study of cardiac remodeling, YAK577 improved heart failure phenotypes in association with suppression of the HDAC8/MMP12 axis. In contrast, in the present vascular smooth muscle cell model, YAK577 preferentially reduced MMP14 expression while MMP12 remained largely unchanged. Although YAK577 was initially characterized as a hydroxamic acid-based HDAC inhibitor with activity toward HDAC8, it is not absolutely isoform-selective and can inhibit additional HDACs to a lesser extent. Therefore, while our findings are consistent with HDAC8-related signaling described in our previous studies, the present data should be interpreted more broadly as reflecting an HDAC-sensitive regulatory network that influences MMP expression and redox-associated calcification pathways. This divergence likely reflects context-dependent HDAC signaling [43,44]. Vascular smooth muscle cells undergoing calcification rely heavily on membrane-associated matrix remodeling programs, in which MMP14 is known to play a prominent role, whereas inflammatory and fibrotic environments such as cardiac remodeling may preferentially engage macrophage-associated proteases including MMP12 [29]. More broadly, epigenetic regulators such as HDACs are increasingly recognized to control distinct transcriptional programs depending on cell type and stimulus [24,25,36]. Within this framework, YAK577 may share a common upstream pharmacologic target but produce different downstream protease signatures depending on the pathological context.

Importantly, the redox data provide a mechanistic bridge between MMP14 and calcification-associated oxidative stress. CM increased ROS as assessed by DHE staining, and both YAK577 and NAC reduced ROS accumulation, supporting oxidative stress as a relevant driver in our in vitro system. YAK577 also suppressed NOX2 upregulation in calcified VSMCs and in vitamin D_3_-treated aortas. This is consistent with previous reports showing that NOX2, together with p22phox and p47phox, is upregulated during vascular calcification and that inhibition or knockdown of this pathway attenuates ROS production and calcification [45]. Notably, MMP14 overexpression increased NOX2, suggesting that MMP14 induction may contribute to a NOX2-associated pro-oxidant state under calcifying conditions. Although direct evidence for a specific MMP14–NOX2 signaling axis remains limited, previous work has shown that MT1-MMP/MMP14 can promote ROS generation and oxidative stress in other cellular systems, supporting the biological plausibility of this connection [46]. Therefore, our findings support a working model in which MMP14 upregulation is associated with increased NOX2-related ROS signaling, which may facilitate activation of osteogenic transcriptional programs and mineral deposition. The stronger NOX2 induction observed after MMP14 overexpression likely reflects a forced gain-of-function condition, whereas the vitamin D_3_ model represents a multifactorial in vivo calcification process. Therefore, these models were not expected to produce quantitatively equivalent changes, and the overexpression experiment was intended mainly to support a directional MMP14–NOX2 relationship. Importantly, our new data further address the apparent discrepancy between the modest change in NOX2 protein abundance and the robust increase in ROS formation and calcification. NOX2-derived ROS production is regulated not only by NOX2 expression but also by activation-dependent assembly of cytosolic subunits. In this regard, CM increased phosphorylation of p47phox at Ser370, total p47phox expression, and the phosphorylated p47phox/total p47phox ratio, whereas YAK577 reduced all of these changes. These findings suggest that calcifying conditions enhance both the abundance and activation state of the NOX2/p47phox system, which may amplify ROS generation beyond what would be predicted from NOX2 protein expression alone. Consistently, pharmacological inhibition of NOX2 with GSK2795039 attenuated CM-induced DHE fluorescence, supporting the contribution of NOX2 activity to ROS accumulation in this model. Therefore, the anti-calcific effect of YAK577 is unlikely to be explained solely by a reduction in NOX2 protein abundance; rather, suppression of p47phox-associated NOX2 activation may represent an additional mechanism by which YAK577 limits oxidative stress and osteogenic reprogramming.

Because YAK577 is an HDAC inhibitor, our results also raise a plausible epigenetic explanation for MMP14 regulation. HDAC inhibition can alter chromatin accessibility and transcriptional output, potentially involving acetylation marks such as H3K9ac or H3K27ac [47,48], linking chromatin remodeling to protease-related transcriptional programs [49]. However, in the absence of locus-specific chromatin evidence, we interpret this as hypothesis-generating and emphasize the need to determine whether MMP14 is directly regulated at the chromatin level or indirectly via upstream signaling pathways.

From a broader perspective, an MMP14–NOX2/ROS-associated axis may be relevant to calcification-prone vascular disease states in which extracellular matrix remodeling and oxidative stress co-exist, including diabetes and chronic kidney disease. However, the in vivo findings should be interpreted within the context of the vitamin D_3_ overload model used here. This model is widely employed because it rapidly and reproducibly induces medial arterial calcification in mice, enabling mechanistic interrogation of calcification in vivo; however, it does not fully recapitulate the chronic and multifactorial biology of human vascular calcification [50]. In addition, vitamin D_3_-driven calcification is often accompanied by systemic disturbances in calcium and phosphate homeostasis, and the relationship between vitamin D_3_ activity and vascular calcification is context-dependent rather than determined by circulating vitamin D_3_ levels alone [51]. Accordingly, our data indicate that YAK577 is active in an established pro-calcific environment, but further studies in chronic disease models and longer-term pharmacological evaluations will be required to determine its translational potential.

## 5. Limitations and Conclusions

Several limitations should be acknowledged. First, we quantified MMP14 abundance but did not directly measure enzymatic activity; activity-based assays would strengthen mechanistic inference. Second, although GSK2795039 reduced CM-induced DHE fluorescence and p47phox phosphorylation supported activation of the NOX2/p47phox axis, additional NOX2-targeted genetic loss-of-function or rescue experiments would further establish NOX2-derived ROS as a required downstream mediator linking MMP14 to osteogenic reprogramming. Third, HDAC isoform selectivity and direct epigenetic regulation of MMP14 were not established and warrant chromatin-focused studies and isoform profiling. In addition, vascular calcification is a complex process, and the contribution of other MMPs or NOX isoforms cannot be excluded. The vitamin D_3_ model used here is an acute induction model that is well suited for studying medial calcification mechanisms in mice, but it does not fully capture the chronic, multifactorial pathobiology of human vascular calcification [51,52].

Despite these limitations, our data support an integrated working model in which YAK577 attenuates vascular calcification through multiple interconnected mechanisms, including suppression of MMP14-associated NOX2-derived ROS signaling and downstream osteogenic reprogramming in VSMCs. These findings suggest that the MMP14–NOX2/ROS axis may represent one component of a broader YAK577-responsive network and provide a rationale for further mechanistic and translational investigation of YAK577.

## Figures and Tables

**Table 1:** Primers used in quantitative real-time polymerase chain reaction analysis.

Genes	Primer Sequence (5′ to 3′)
Actb (mouse_NM_007393.5)	F:CCTCTATGCCAACACAGTGC R:CCTGCTTGCTGATCCACATC
*Bmp2* (rat_NM_017178.2)	F: TACCCCCGGCTGTGATGCGA R: ACCCGCAACCCTCCACAACC
*Runx2* (rat_NM_001278483.2)	F: GAGCACAAACATGGCTGAGA R: TGGAGATGTTGCTCTGTTCG
*Msx2* (rat_NM_012982.3)	F: AACACAAGACCAACCGGAAG R: GCAGCCATTTTCAGCTTTTC
*Mmp1* (rat_NM_001134530.1)	F: CTTGCTCACACATTCCCACC R: AGCTGGGGAACATTAGTGCT
*Mmp2* (rat_NM_031054.2)	F: ACACAAACACGATATGGACCTA R: ACACAAACACGATATGGACCTA
*Mmp3* (rat_NM_133523.3)	F: ATGACAGGGAAGCTGGACTC R: CTGGAGAATGTGAGTGGGGT
*Mmp7* (rat_NM_012864.2)	F: GTTGATGGCAGCTATGAGGC R: CTTTCCAGTCTCCGGCAAAC
*Mmp9* (rat_NM_031055.2)	F: AGGATGGTCTACTGGCACAC R: GTGCAGGACAAATAGGAGCG
*Mmp10* (rat_NM_133514.2)	F: TGGAGATGACAGGGAAGCTG R: CCTCCTCCCAGACCTTCAAA
*Mmp12* (rat_NM_053963.2)	F: TGCAGCTGTCTTTGATCCAC R: TCCAATTGGTAGGCTCCTTG
*Mmp14* (rat_NM_031056.1)	F: ATGGAAGCAAGTCAGGGTCA R: ACCATCGCTCCTTGAAGACA
*Gapdh* (mouse_NM_001411840.1)	F: GGGTCCCAGCTTAGGTTCAT R: CATTCTCGGCCTTGACTGTG
*Bmp2* (mouse_NM_007553.3)	F: TCCCCAGTGACGAGTTTCTC R: CGAAGCTCTCCCACTGACTT
*Runx2* (mouse_NM_001145920.3)	F: GCCCAGGCGTATTTCAGATG R: GGTAAAGGTGGCTGGGTAGT
*Msx2* (mouse_NM_013601.2)	F: TTCACCACATCCCAGCTTCT R: TTCAGCTTTTCCAGTTCCGC
*Mmp2* (mouse_NM_008610.3)	F: ATGACATCAAGGGGATCCAG R: GTCACGTGGTGTCACTGTCC
*Mmp3* (mouse_NM_010809.3)	F: CAGTGCAAGGGATGATGATG R: CATCAGGAACACCACACCTG
*Mmp7* (mouse_NM_010810.6)	F: GGAGACAGCTTCCCCTTTGA R: CCGGGAACAGAAGAGTGACT
*Mmp9* (mouse_NM_013599.5)	F: GAAGGCAAACCCTGTGTGTT R: AGGAAGACGAAGGGGAAGAC
*Mmp10* (mouse_NM_019471.3)	F: TCTGCTCAGCGTATCCTCTG R: TTCCCTGTCATCTCCAACCC
*Mmp12* (mouse_NM_008605.3)	F: CTGGTTCTTCTGGTGGAAGC R: ATGCTCCTGGGATAGTGTGG
*Mmp13* (mouse_NM_008607.2)	F: CCAGAACTTCCCAACCATGT R: GTCTTCCCCGTGTTCTCAAA
*Mmp14* (mouse_NM_008608.4)	F: AACATCCATCCCGTGACCTT R: TTCTCAAAGTGAACCGCAGC

## Data Availability

The original contributions presented in this study are included in the article/Supplementary Materials. Further inquiries can be directed to the corresponding authors.

## Supplementary Materials

The following supporting information can be downloaded at: https://www.mdpi.com/article/10.3390/antiox15050605/s1, Figure S1: YAK577 mitigates MMP expression in vitamin D_3_-treated mice (A–H).
