# Supplementary material for: YAK577 Attenuates Vascular Calcification by Targeting an MMP14–NOX2/ROS Axis in VSMCs and a Vitamin D3-Induced Mouse Model
Source: Antioxidants (Basel). 2026 May 10;15(5):605. doi: 10.3390/antiox15050605 (PMC13203591; doi:10.3390/antiox15050605)

Supplementary Figure S1

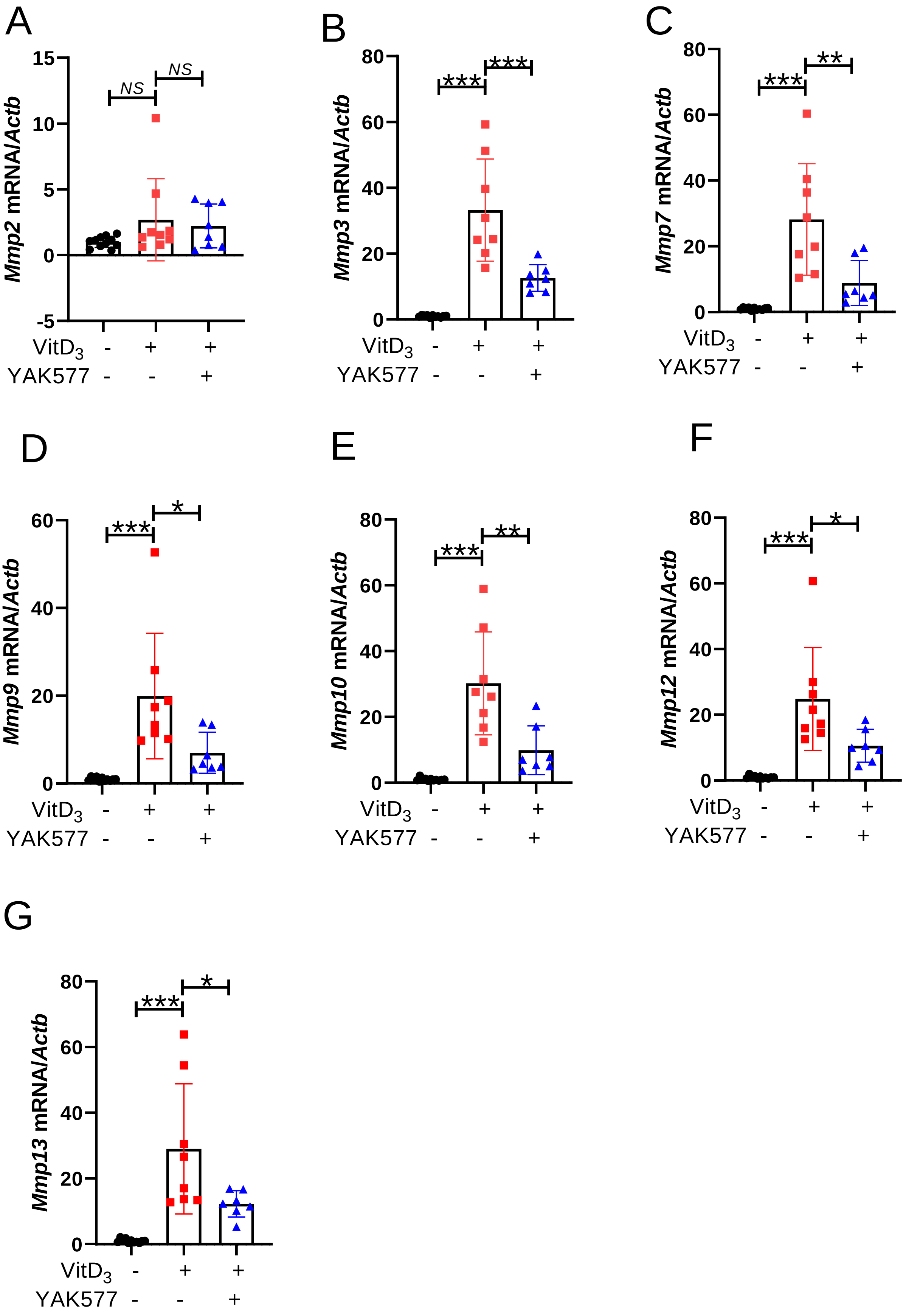

Supplementary Figure S1. YAK577 mitigates MMP expression in vitamin D_3-treated mice.

(A–G) The mRNA levels of *Mmp2*, *Mmp3*, *Mmp7*, *Mmp9*, *Mmp10*, *Mmp12*, and *Mmp13* in vitamin D_3-treated mice with or without YAK577. Each mRNA levels were normalized to the β-actin (*Actb*). Data are presented as mean ± SEM. *** $p < 0.001$; ** $p < 0.01$; * $p < 0.05$. Each dot represents one independent biological experiment (not a technical replicate).

Figure 2 C: Western blot (original gels)

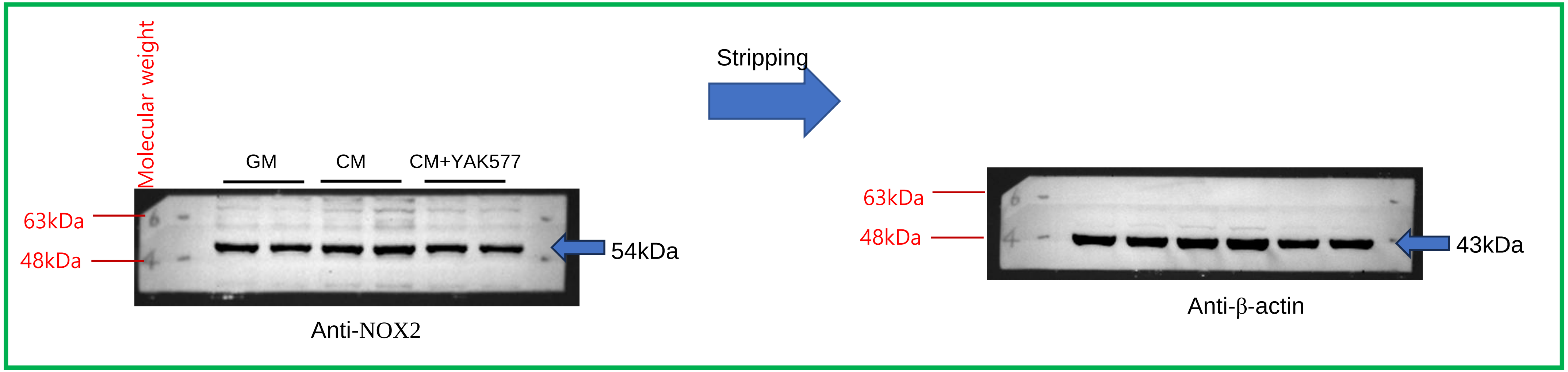

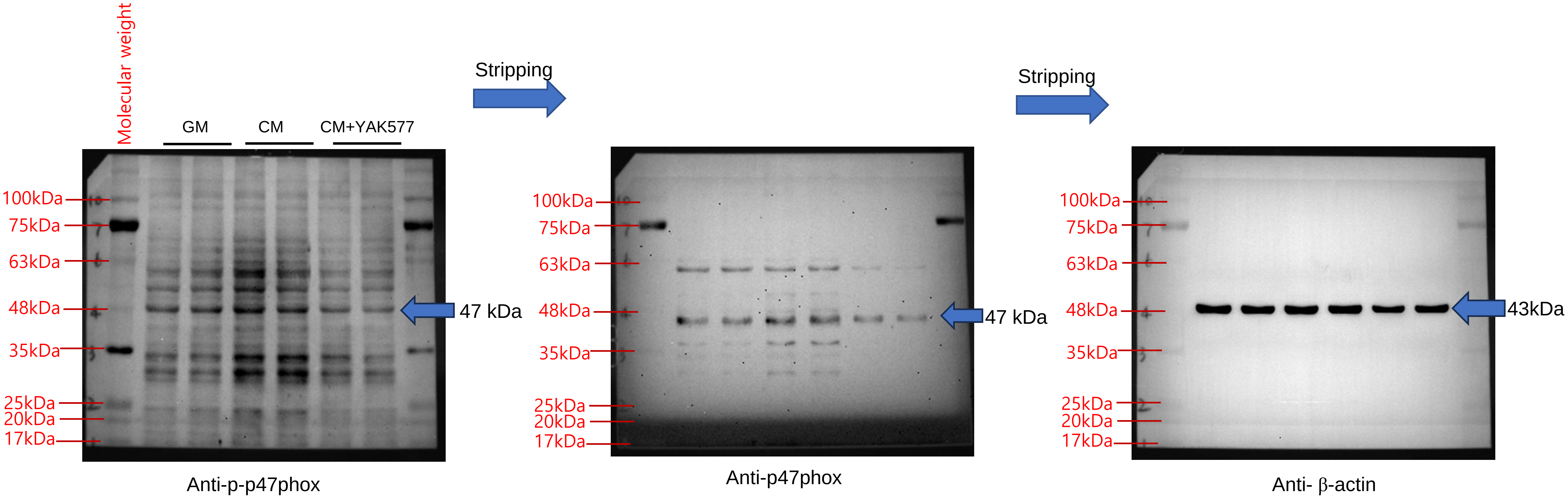

Figure 3D: Western blot (original gels)

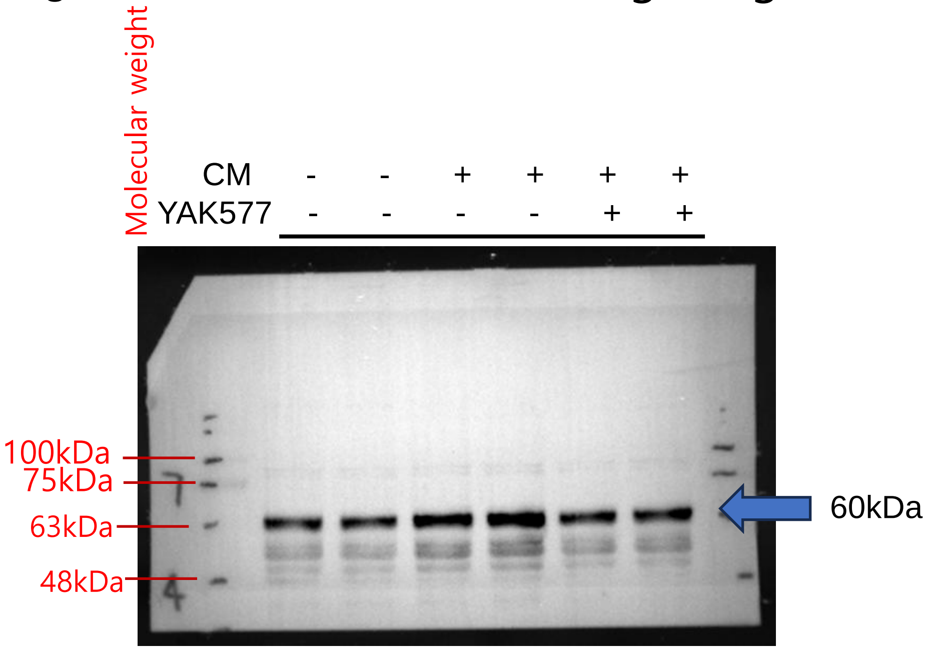

Anti-RUNX2

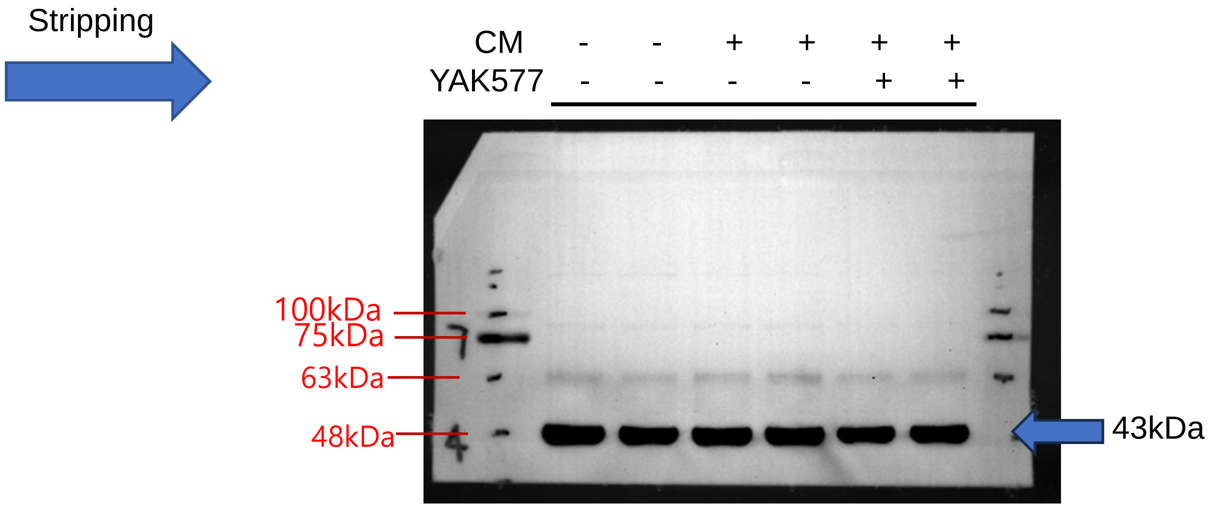

Anti-β-actin

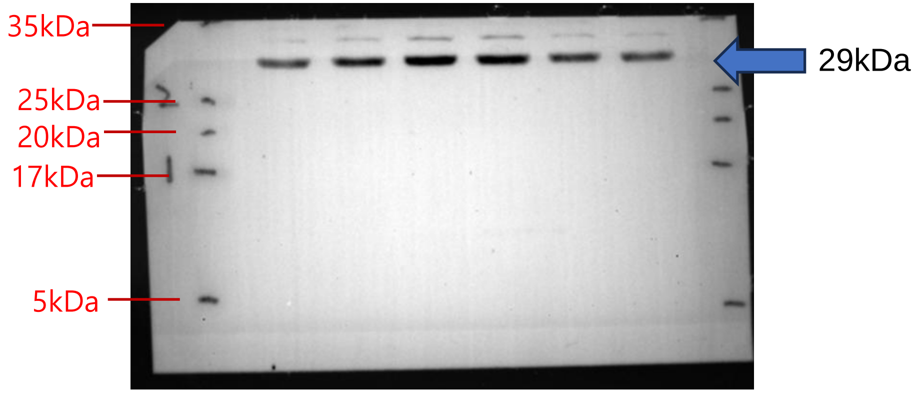

Anti-MSX2

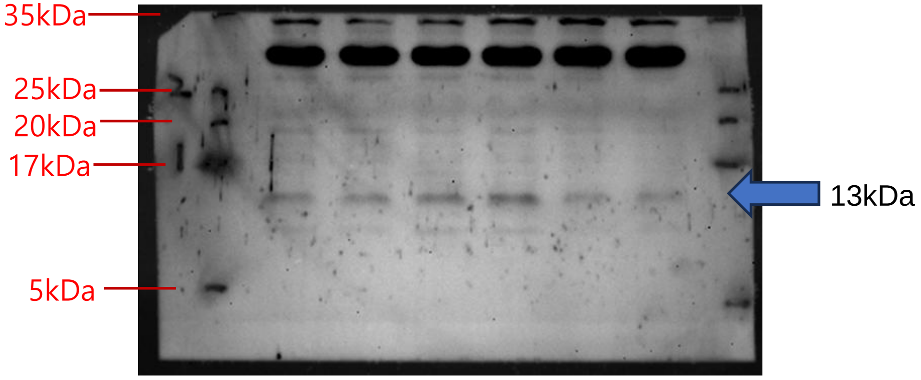

Anti-BMP2

Figure 4 I: Western blot (original gels)

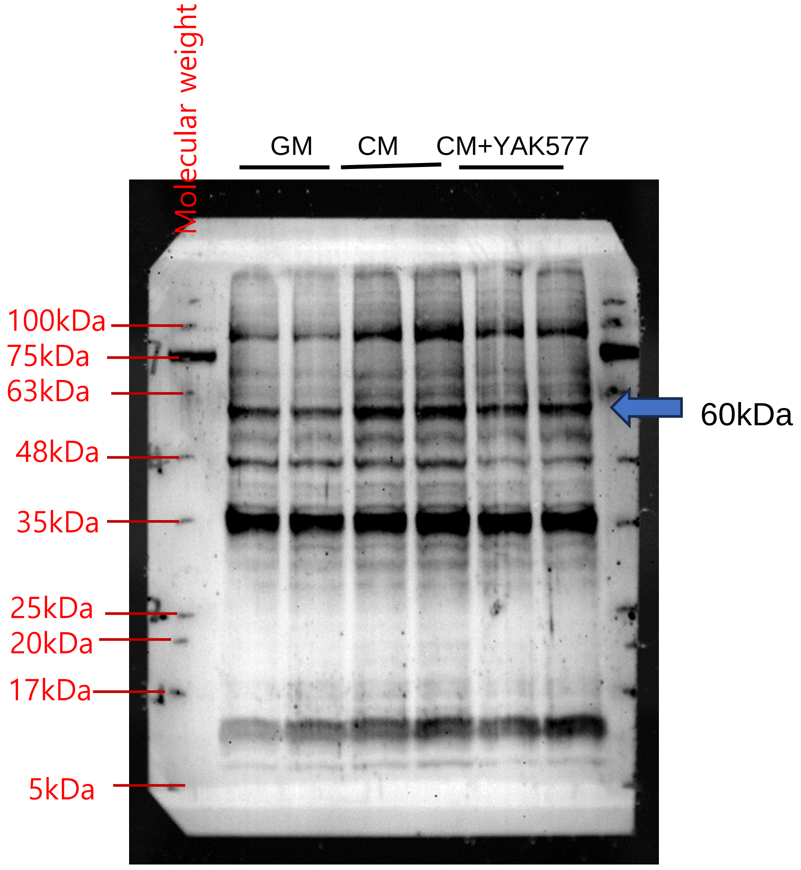

Anti-MMP14

Stripping

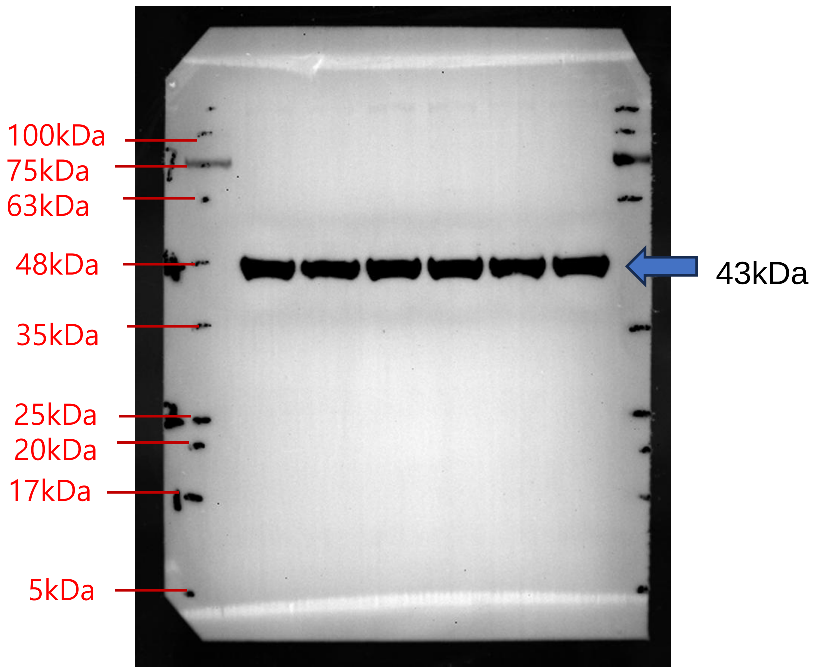

Anti-β-actin

Figure 6 E: Western blot (original gels)

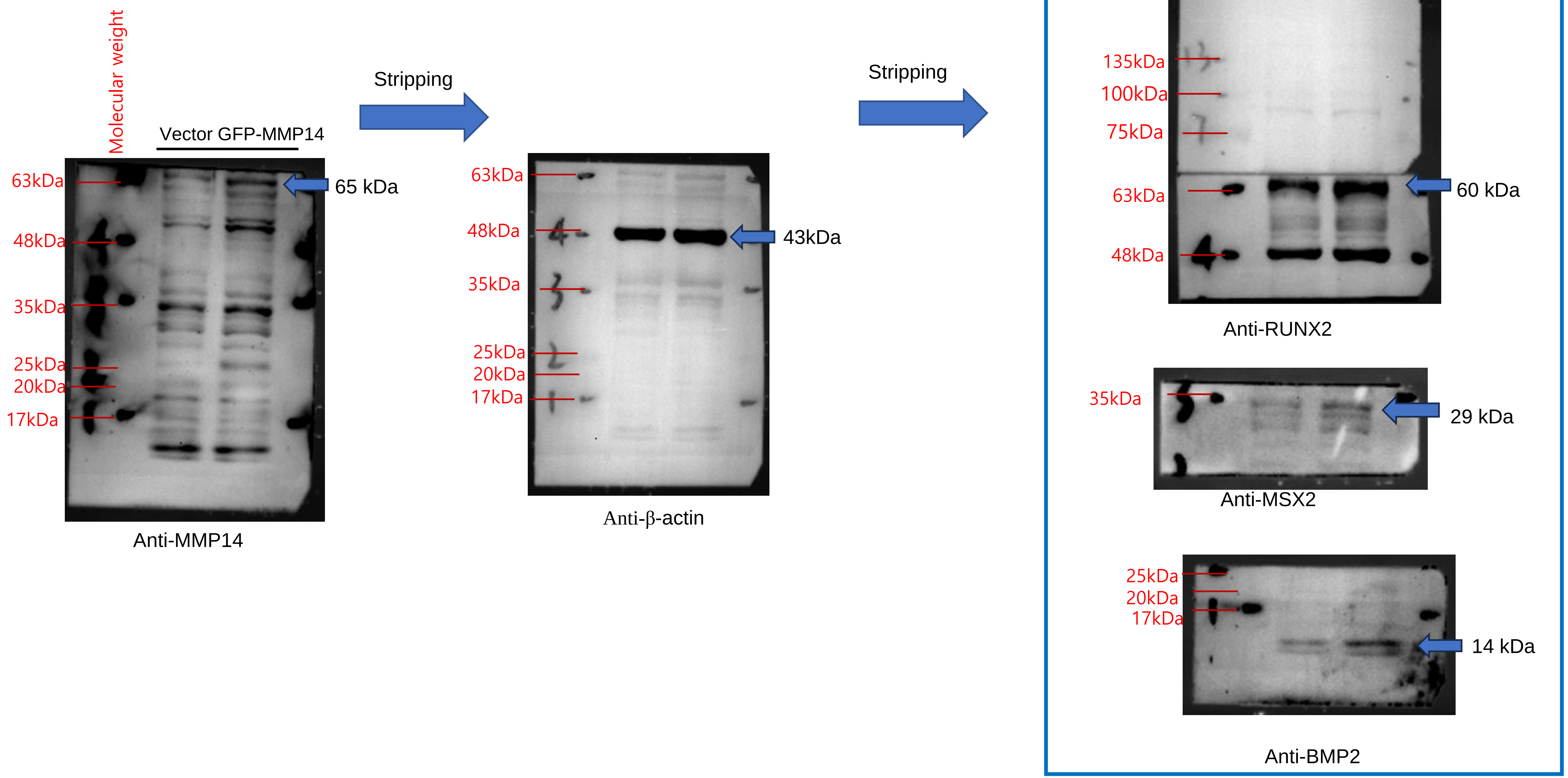

Figure 6 E: Western blot (original gels)

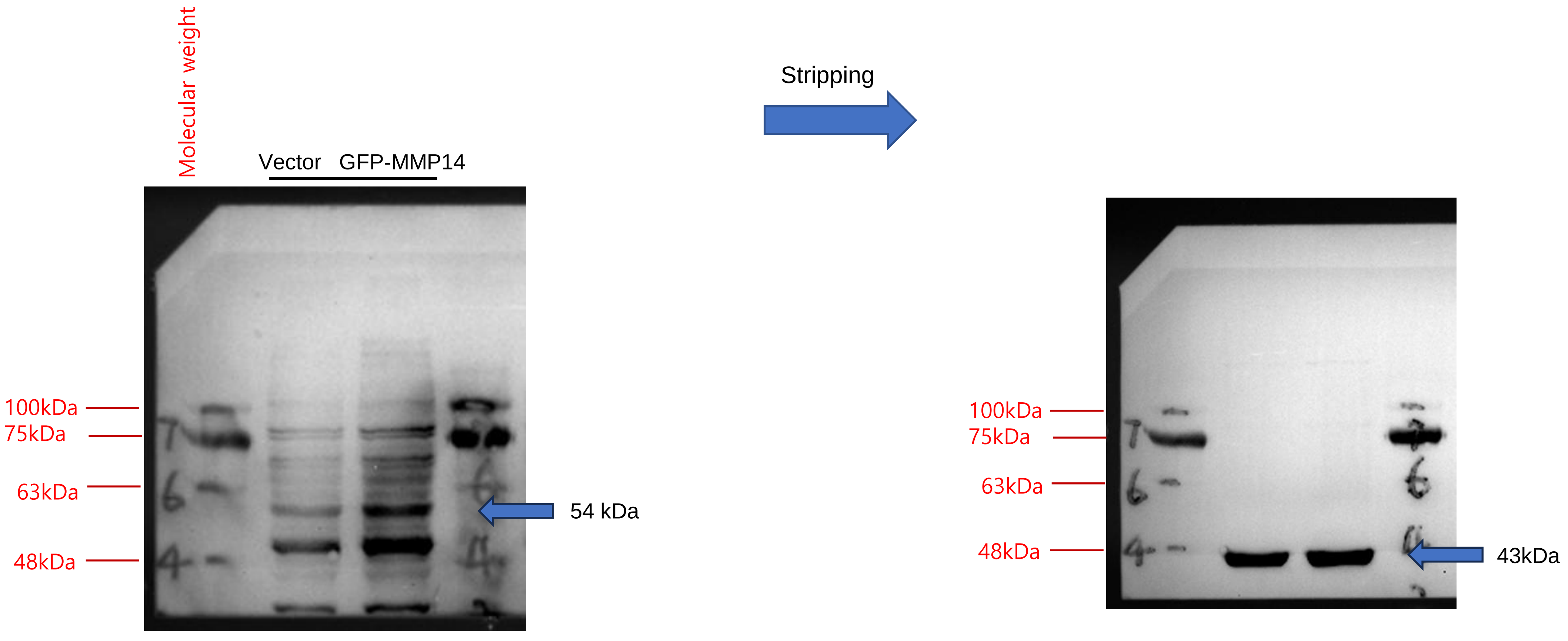

Anti-NOX2

Anti-β-actin

Figure 7 G Western blot (original gels)

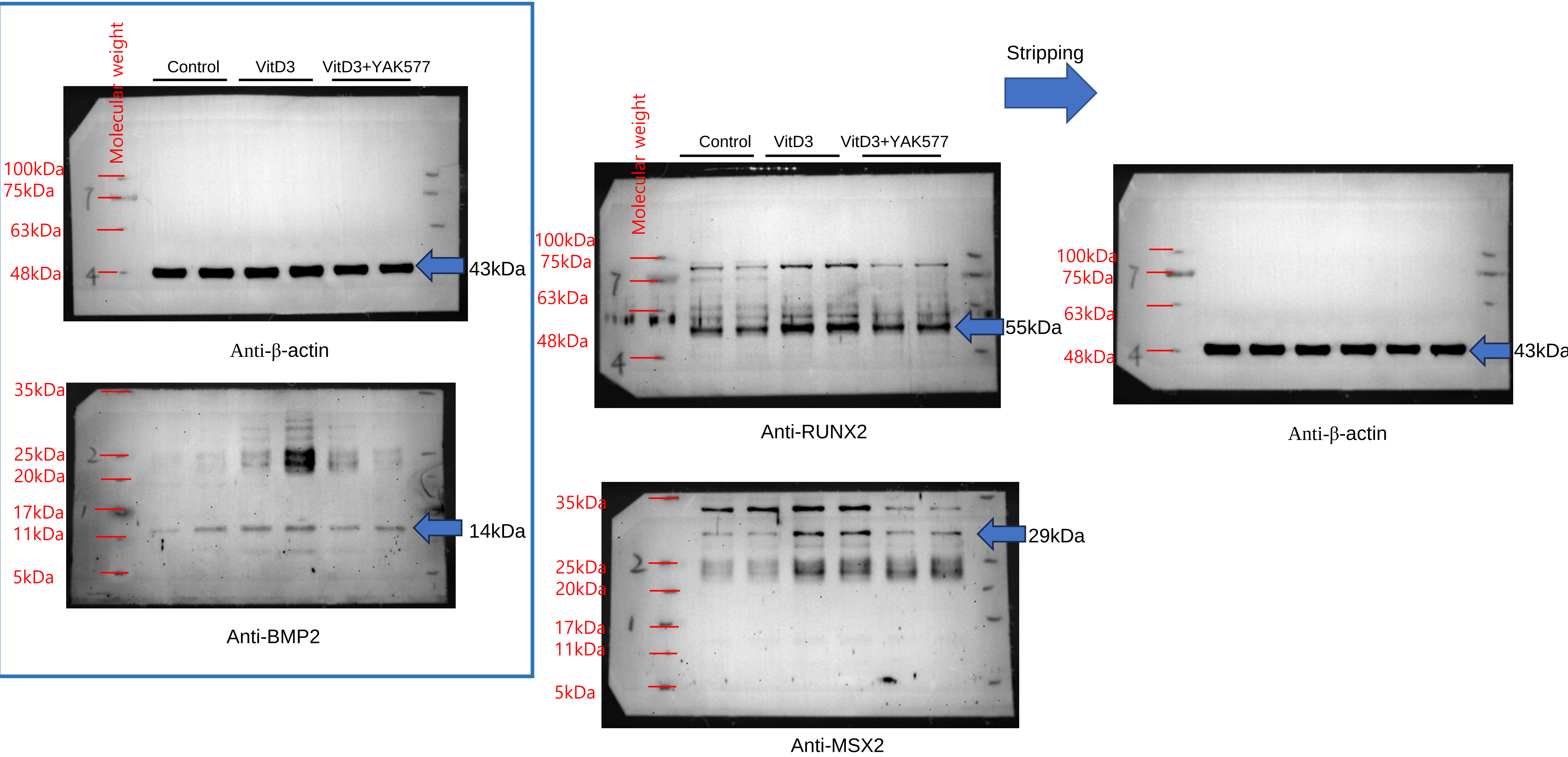

Figure 8 B: Western blot (original gels)

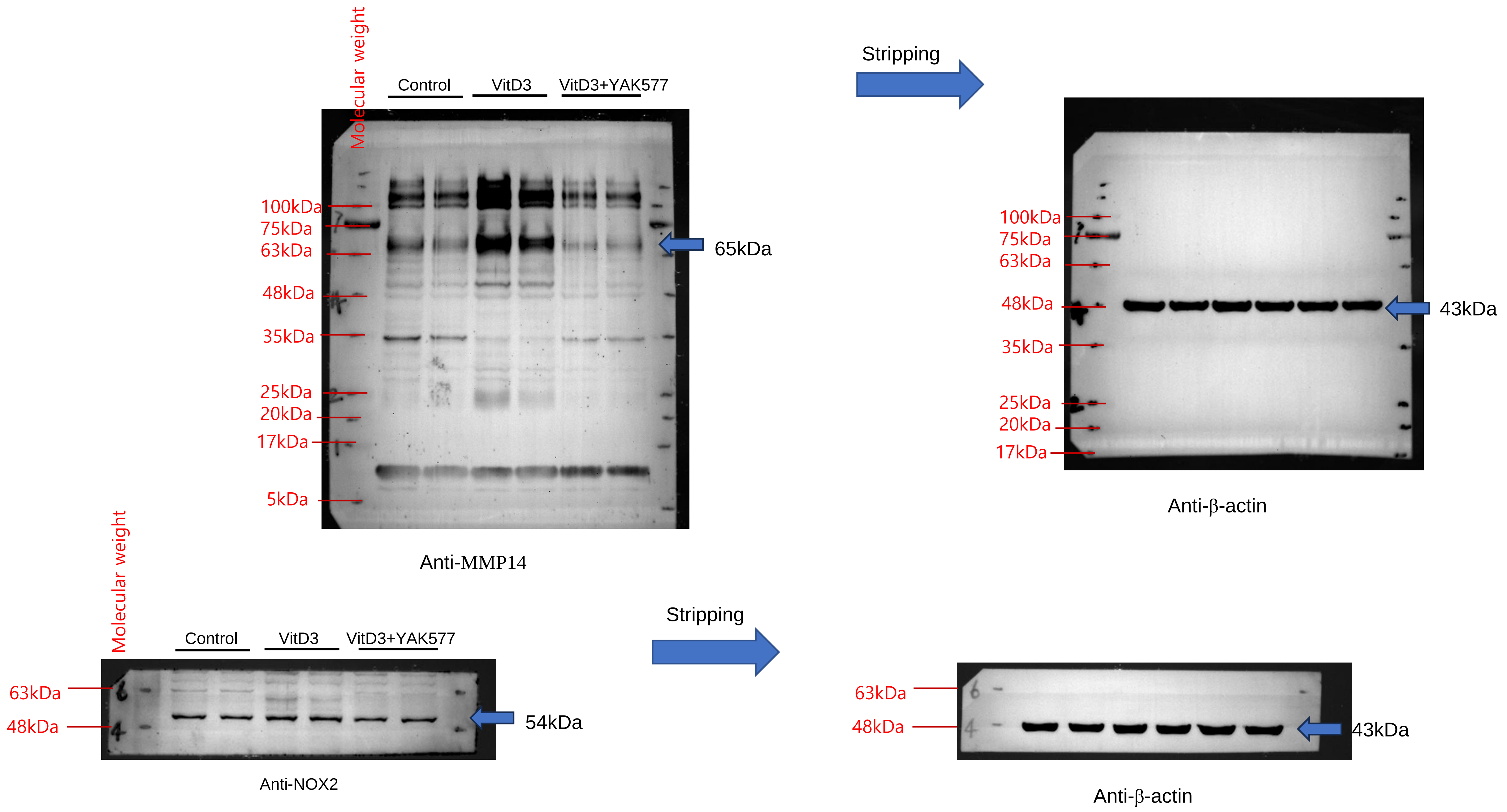

Supplement: Supplementary file 1 [file antioxidants-15-00605-s001.zip › antioxidants-4180704-supplementary.pdf]
